# Causal association between blood metabolites and head and neck cancer: butyrylcarnitine identified as an associated trait for cancer risk and progression

**DOI:** 10.1186/s41065-025-00408-5

**Published:** 2025-03-14

**Authors:** Ying Li, Zihan Chen, Zongwei Huang, Jing Wang, Jue Wang, Lanxin Lin, Ruyu Lin, Jinghua Lai, Libin Zhang, Sufang Qiu

**Affiliations:** 1 Clinical Oncology School of Fujian Medical University, Fujian Cancer Hospital, Fuzhou, 350014 Fujian China; 2 Fujian Medical University, Fujian, China; 3 Fujian Key Laboratory of Translational Cancer Medicine, Fujian, China; 4 Fujian Provincial Key Laboratory of Tumor Biotherapy, Fujian, China

**Keywords:** Head and neck cancer, Causal relationship, Butyrylcarnitine, Cell proliferation, Mendelian randomization

## Abstract

**Background:**

Blood metabolites play an important role in predicting or influencing the occurrence and development of cancers. We aimed to evaluate the relationship between blood metabolites and the occurrence of head and neck cancer (HNC).

**Methods:**

We employed a Mendelian randomization (MR) approach to investigate the role of blood metabolites in HNC predisposition. The HNC cell line HN30 was treated with butyrylcarnitine, the metabolite identified through MR analysis, and subjected to a series of cellular assays to assess its potential carcinogenic effects.

**Results:**

Among the 258 blood metabolites analyzed, butyrylcarnitine emerged as the only metabolite demonstrating a potential causal association with HNC risk following Bonferroni correction (inverse-variance-weighted MR method: β = 0.904, *P* < 0.001). Genetically predicted higher levels of butyrylcarnitine (log-transformed) were causally linked to an increased risk of HNC (OR: 2.470, 95% CI: 1.530–3.987). Sensitivity analyses, including MR-Egger regression, leave-one-out analysis, and funnel plots, confirmed the robustness of the findings, with no evidence of directional pleiotropy. In vitro experiments further demonstrated that butyrylcarnitine promoted the proliferation, migration and invasion of HN30 cells.

**Conclusions:**

By employing a genetic epidemiological framework, our research assessed the impact of metabolite butyrylcarnitine on HNC susceptibility. These findings offer valuable insights into potential therapeutic targets and highlight the promise of targeted metabolic strategies for reducing HNC risk. Nevertheless, further research is required to elucidate the precise biological mechanisms underlying these findings.

**Supplementary Information:**

The online version contains supplementary material available at 10.1186/s41065-025-00408-5.

## Introduction

Head and neck cancer (HNC) represents the sixth most common malignancy worldwide, reporting over 890,000 newly diagnosed cases and approximately 450,000 fatalities in the year 2020 [1]. Significant advancements in imaging, diagnostic techniques, and therapeutic modalities have contributed to improved survival rates among HNC patients [2, 3]. Oropharyngeal cancer, the most frequently occurring subtype of HNC, is primarily associated with well-recognized risk factors such as tobacco use and alcohol consumption [4]. Moreover, epidemiological studies reveal a notable upward trend in the incidence of HNC, with an average annual rise of 2.9% over the last decade, which is largely attributed to the heightened prevalence of human papillomavirus (HPV) infection, that has also emerged as a significant factor in the development of HNC [4, 5]. Despite efforts to mitigate risk factors such as tobacco and alcohol use, as well as mass prophylactic HPV vaccination, the burden of HNC continues to persist at substantial levels [6]. Consequently, exploring the pathogenesis underlying HNC and identifying additional modifiable risk factors and preventive targets are of great importance for advancing strategies in HNC prevention and treatment.

Human blood metabolites, the end products of metabolic processes within the bloodstream, serve as dynamic indicators of both intrinsic physiological states and extrinsic environmental influences, providing a holistic representation of biological activity [7]. As functional intermediates under environmental exposures, these metabolites often mirror an individual’s genetic composition and have the potential to predict or influence the onset and progression of diseases [8]. Recently, studies on blood metabolites have provided numerous biomarkers and established reliable prediction models for predicting tumors, including breast, prostate, and kidney cancers, among others [9]. However, the causal relationships between blood metabolites and HNC remain poorly understood.

Mendelian randomization (MR) is a method that utilizes genetic variants as instrumental variables (IVs) to infer causal relationships between risk factors and disease outcomes [10–12]. In contrast to traditional observational studies, MR is less susceptible to confounding biases and reverse causality [13]. Metabolomic-based genome-wide association studies (GWASs) are an effective way for identifying quantitative trait loci associated with metabolites, thereby elucidating the metabolic context of disease-related genetic variations and providing novel insights into their causal links with cancer [14]. In 2014, Shin et al. conducted one of the largest GWASs on human blood metabolites to date, offering valuable perspectives into the genetic architecture of blood metabolomics [15]. Recently, Smith-Byrne et al. applied bidirectional MR analysis to investigate the causal relationship between blood metabolites and lung cancer, identifying an inverse association between elevated blood isovalerylcarnitine levels and lung cancer risk [16]. Similarly, Zhong et al. demonstrated a link between metabolite X-21,849 and pancreatic ductal adenocarcinoma risk, while Wang et al. highlighted the mediating role of high-density lipoprotein cholesterol and acetate in breast cancer development [17, 18]. A prior MR study evaluated the effects of obesity, blood glucose, and blood pressure traits, in conjunction with circulating lipid metabolites, on oropharyngeal cancer risk [19]. However, limited evidence exists to support a causal association between genetically predicted metabolic traits and the development of HNC.

In this study, we employed a two-sample MR approach to systematically investigate the causal relationships between 258 blood metabolites and the risk of HNC, with butyrylcarnitine identified as a metabolite of interest. To further explore its potential role in HNC pathogenesis, we conducted in vitro experiments to validate the oncogenic effects of butyrylcarnitine in HNC cell lines. These findings provide valuable insights for advancing risk prediction strategies and identifying potential therapeutic targets for HNC.

## Methods

### Study design

**Fig. 1:**
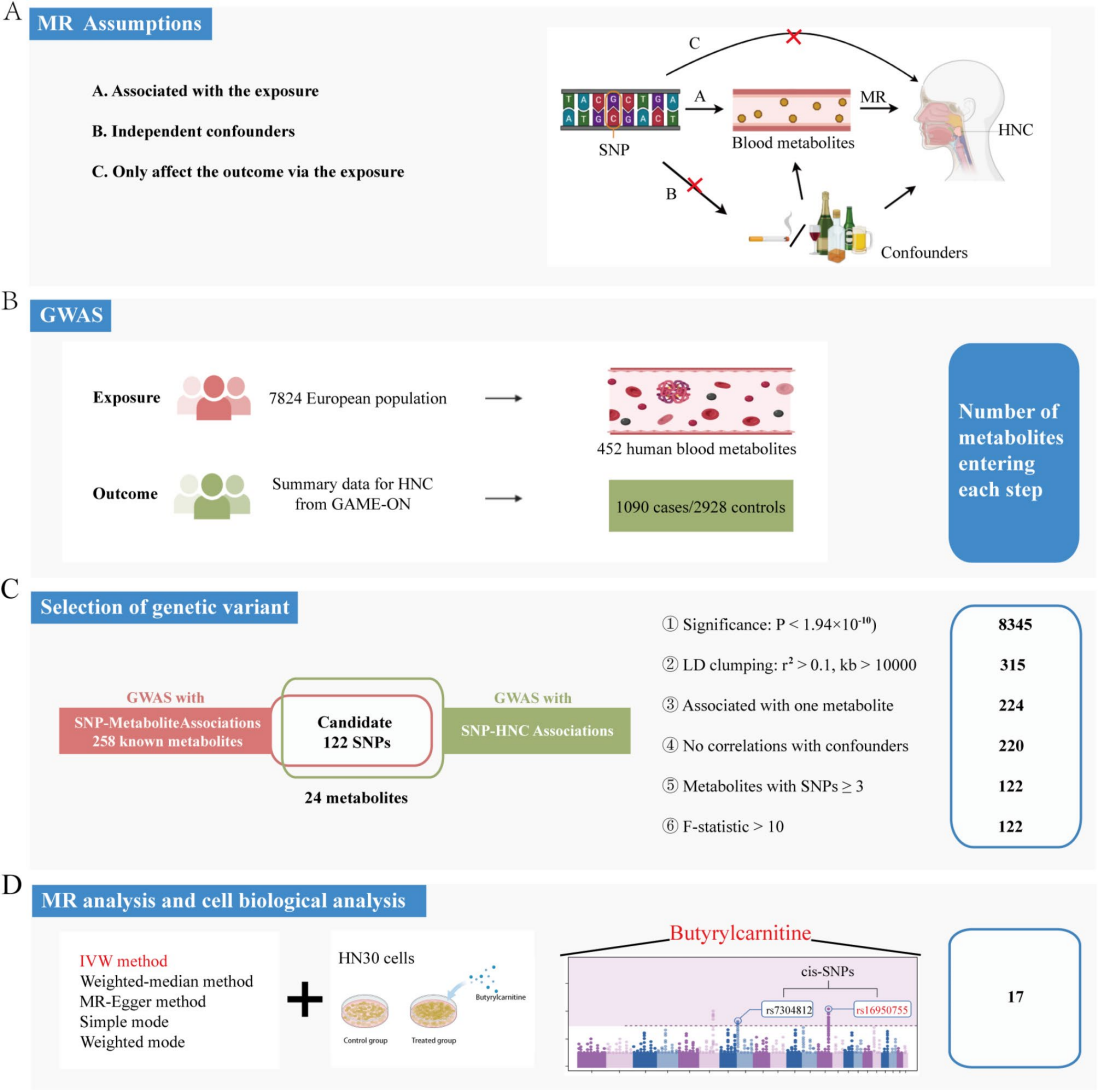
Description of overall study design. (**A**) The causal interpretation of MR estimates relies on 3 assumptions: independence, relevance, and exclusivity; (**B**) The data used in this study including a GWAS summary statistic for blood metabolomic traits and summary data for HNC (from GAME-ON); (**C**) The selection of genetic variant including: Step 1, SNPs with a genome wide significance level (a stringent cutoff: *P* < 1.94 × 10^–10^, 5 × 10^− 8^/258 metabolites); Step 2, adjust LD: set distance apart from each other > 10,000 kb and r^2^ > 0.1; Step 3, excluded SNPs associated with more than one metabolite; Step 4, excluded SNPs which have genetic correlations with known confounders of HNC; Step 5–6, remaining IVs with 3 or more valid genetic variants and F-statistic > 10; (**D**) The IVW method, weighted-median method, MR-Egger method, simple mode and weighted mode were employed to give consistent association estimates. Only one metabolite, butyrylcarnitine, remained associated after these analyses, with no heterogeneity and directional pleiotropy following sensitivity analyses, including Cochran’s Q test and MR-Egger regression method; And then the HNC cell line HN30 was exposed to metabolite butyrylcarnitine and subjected to cell biological analyses to explore the possible carcinogenic effects of butyrylcarnitine in HNC; GAME-ON, Genetic Associations and Mechanisms in Oncology; GWAS, genome-wide association study; HNC, head and neck cancer; IVW, inverse-variance-weighted method; LD, linkage disequilibrium; MR, Mendelian randomization; SNPs, single nucleotide polymorphisms

To investigate the causality of human blood metabolites (exposure) on the risk of HNC (outcome), we conducted a two-sample MR analysis. The fundamental tenets of MR analysis, namely relevance, independence, and exclusion restriction, were upheld throughout this investigation, meaning (1) the selected genetic variants are robustly associated with the exposure; (2) the genetic variants remain independent of potential confounding factors; and (3) their influence on the outcome was mediated exclusively through the exposure. Furthermore, our study was restricted to individuals of European ancestry to mitigate potential biases arising from population stratification. The analysis utilized publicly available data from GWASs, all of which had obtained approval from relevant ethical review committees. The study adhered to the STROBE-MR guidelines [20]. A schematic overview of the study design is presented in Fig. 1.

### Summary statistics of explore and outcome

In this study, the GWAS summary statistics were obtained from the Integrative Epidemiology Unit (IEU) open GWAS project (https://gwas.mrcieu.ac.uk/), which aggregates and analyzes GWAS data from sources including the UK Biobank, FinnGen biobank, and published literature. We accessed GWAS summary statistics for 452 available blood metabolomic traits (GWAS ID: met-a), derived from a meta-analysis of up to 7824 individuals of European ancestry across three independent cohorts: two TwinsUK cohorts, and Cooperative Health Research in the Region of Augsburg (KORA) study [15, 21, 22]. This GWAS is a large and comprehensive meta-analysis of aggregated genetic data on human metabolism, with detailed participant characteristics and metabolite annotations described by Shin et al. [15]. Among these, 258 known metabolites, categorized into 8 major groups (amino acid, lipid, carbohydrate, energy, nucleotide, cofactor and vitamin, peptide, and xenobiotic metabolism), were included in further analyses. Summary data for HNC were derived from a GWAS comprising 1090 HNC cases and 2928 controls of European ancestry, identified using International Classification of Diseases (ICD) codes: C01.9, C02.4, and C09.0-C10.9 [23]. Detailed information on participant demographics, genotyping methods, and imputation techniques is available in prior publications [24]. Additional details regarding the datasets used in this study are provided in Supplementary Table 1.

### Selection of genetic variant

This study initially employed a genome-wide significance threshold of *P* < 5 × 10^− 8^ to identify single nucleotide polymorphisms (SNPs) independently associated with blood metabolite levels [25]. To account for multiple testing, a Bonferroni correction was implemented, resulting in a more stringent significance threshold of *P* < 1.94 × 10^− 10^ (5 × 10^− 8^/258 metabolites). We set a distance of > 10,000 kb between SNPs and adjusted for linkage disequilibrium (LD) (defined as r^2^ > 0.1 in European populations) to ensure the selection of conditionally independent IVs [26]. The proportion of variance in the exposure variable explained by each SNP was quantified using R^2^ [27]. The strength of individual SNPs was validated using the F-statistic, calculated as the square of the beta coefficient for the exposure divided by its variance, with an F-statistic > 10 indicating sufficient strength to minimize potential bias [28]. Detailed methods for calculating R^2^ and the F-statistic have been described elsewhere [27, 28]. To assess potential genetic correlations between SNPs and HNC, we queried the PhenoScanner database (version 2, http://www.phenoscanner.medschl.cam.ac.uk/) [29]. Summary statistics for associations with HNC were extracted for IVs comprising 3 or more valid genetic variants. We finally leaved 122 SNPs associated with 24 human blood metabolites as IVs for the subsequent MR analyses.

### Mendelian randomization analysis

A harmonization process was implemented to ensure the consistency in the effect directions in associations between exposure and outcome variables. Potentially palindromic SNPs were excluded from the analysis if their minor allele frequencies (MAFs) were below 0.3 or if alignment was ambiguous. SNPs serving as IVs would be omitted if they were missing from the outcome summary statistics. The inverse-variance-weighted (IVW) method was applied as the primary analytical approach to assess the potential causal effects of circulating blood metabolites on the risk of HNC [30]. To ensure robustness, alternative methods were also utilized, including the weighted-median method, MR-Egger regression, simple mode, and weighted mode [31–34]. Heterogeneity among the SNPs was assessed using Cochran’s Q test. A random-effects model was applied in cases of significant heterogeneity, while a fixed-effects model was used when no heterogeneity was detected [35]. Additionally, a “leave-one-out” analysis was performed to assess for the stability of the results by deleting each SNP in turn and re-running the MR analysis using the IVW approach with the remaining SNPs. And then the funnel plots and forest plots were generated. A positive finding was considered if the IVW method demonstrated statistical significance with no evidence of pleiotropy or heterogeneity, even if alternative methods yielded non-significant results, provided that their beta values were consistent in direction. Additionally, the MR Steiger test was performed to assess potential reverse causality [36]. The Functional Mapping and Annotation of Genome-wide Association Studies (FUMA GWAS) online tool (https://fuma.ctglab.nl/) was applied to explore the potential biological background of the findings [37].

### Cell cultures and reagents

The human HNC cell line HN30 employed in this study was purchased from MeisenCTCC company (China). Cells were cultured in DMEM (Shanghai BasalMedia Technologies, China), supplemented with 10% fetal bovine serum (FBS, Gibco, USA), 1% penicillin-streptomycin-amphotericin B solution (Biosharp, China), and maintained at 37℃ in a humidified incubator with 5% CO_2_. Butyrylcarnitine (catalog #HY-113168) was obtained from MedChemExpress (USA) and subsequently dissolved in DMEM to prepare a stock concentration of 10 mM.

### Working concentration screening

Exponentially proliferating cells were seeded at a density of 10,000 cells per well in 100 µL of culture medium within 96-well plates and incubated for 24 h. The cells were then treated with varying concentrations of butyrylcarnitine (0, 1 nM, 10 nM, 100 nM, 1 µM, 10 µM, 100 µM, 1 mM, and 10 mM) for 72 h. Following treatment, the culture medium was removed, and 100 µL of fresh medium containing 10% Cell Counting Kit-8 (CCK-8) solution (MedChemExpress, USA) was added to each well. The plates were incubated for an additional 2 h, after which the optical density at 450 nm was measured using a microplate reader (Tecan Infinite 200pro, Switzerland).

### Cell proliferation assay

Cell proliferation was assessed using both the CCK-8 and colony formation assays. For the CCK-8 assay, cells were seeded into 96-well plates at a density of 4,000 cells per well. Following a 24-hour incubation period, the cells were treated with the butyrylcarnitine working solution and subsequently incubated with 10% CCK-8 reagent for 2 h at time points 0, 24, 48, 72, and 96 h post-treatment. Absorbance was measured at 450 nm using a microplate reader, and the data were analyzed to quantify cell viability. For the colony formation assay, cells were seeded in 6-well plates at 4,000 cells per well and cultured for 10 days to allow colony formation. The resulting colonies were then counted and analyzed to assess proliferative capacity.

### Wound healing assay

Cells were plated in 24-well plates at 70–80% confluency and cultured for 24 h in a humidified incubator to achieve full confluence. Once a confluent monolayer was formed, a scratch was made across the cell layer using a sterile pipette tip, followed by washing with phosphate-buffered saline (PBS, Gibco, USA) to remove detached cells and debris. Cells were left untreated (controls) or exposed to butyrylcarnitine. Images of the wound area were captured at 0, 8, and 16 h post-scratch, and the wound closure rate was quantified by measuring the average wound area using ImageJ software.

### Cell migration and invasion assay

Cell migration was evaluated using Transwell chambers (8 μm, Corning, USA). Cells were seeded in the upper chamber at a density of 1 × 10^5^ cells per chamber in serum-free DMEM. The lower chamber was filled with DMEM supplemented with 20% FBS, with or without butyrylcarnitine, to act as a chemoattractant. After a 72-hour incubation at 37 ℃, non-migratory cells remaining in the upper chamber were removed using cotton swabs. Migratory cells in the lower chamber were then fixed with formaldehyde (Beyotime Biotech Inc., Shanghai, China) for 15 min and stained with 0.1% crystal violet (Beyotime Biotech Inc., Shanghai, China) for 15 min. Five randomly selected fields were imaged, and the number of positively stained cells was counted using a microscope. For the invasion assay, the upper chamber was pre-coated with Matrigel (Corning, USA), diluted 1:8 with serum-free cell medium, and incubated at 37 ℃ for the Matrigel to solidify. The subsequent steps followed the same protocol as the migration assay.

### Statistical analysis

All experiments were repeated three times. Data are presented as the mean ± standard deviation (SD). Statistical analyses were conducted using Student’s t-tests in GraphPad Prism 8 software, with *P* < 0.05 considered statistically significant. R software assisted in performing other statistical analysis (v4.0.4, https://www.r-project.org/). MR analysis was conducted using the “TwoSampleMR” package [36]. Associations with *P* values < 0.002 (*P* = 0.05/24) were considered statistically significant after Bonferroni correction for 24 metabolites analyzed in MR analysis. Associations with *P* values < 0.05 but above the Bonferroni correction threshold were regarded as suggestive evidence of a potential causal relationship.

## Results

### Selection of the genetic instruments for blood metabolites

This study chose human blood metabolites as exposures to performed a two-sample MR analysis for HNC. Among the 452 available blood metabolomic traits, 8345 SNPs meeting genome-wide significance (*P* < 1.94 × 10^− 10^) were associated with 258 metabolites of known classification. Following linkage disequilibrium analysis, 315 independent SNPs were identified as genetic predictors (Supplementary Table 2). Of these, 37 SNPs associated with more than one metabolite and 4 SNPs with genetic correlations to known HNC confounders were excluded (Supplementary Table 3). After further excluding metabolites with fewer than 3 SNPs, a total of 122 independent SNPs were selected as IVs, representing 24 human blood metabolites categorized into 6 groups: amino acid, lipid, cofactor and vitamin, energy, nucleotide, and peptide. The number of SNPs for each metabolite is illustrated in Supplementary Fig. 1. The SNPs for each blood metabolite explained ~ 6.58% of the phenotypic variance, and the F-statistic values surpassed the threshold of 10, thereby signifying low susceptibility to weak instrument bias. After data harmonization, 102 valid genetic variants were retained for analysis.

### Genetically determined metabolite butyrylcarnitine and risk of HNC

**Fig. 2:**
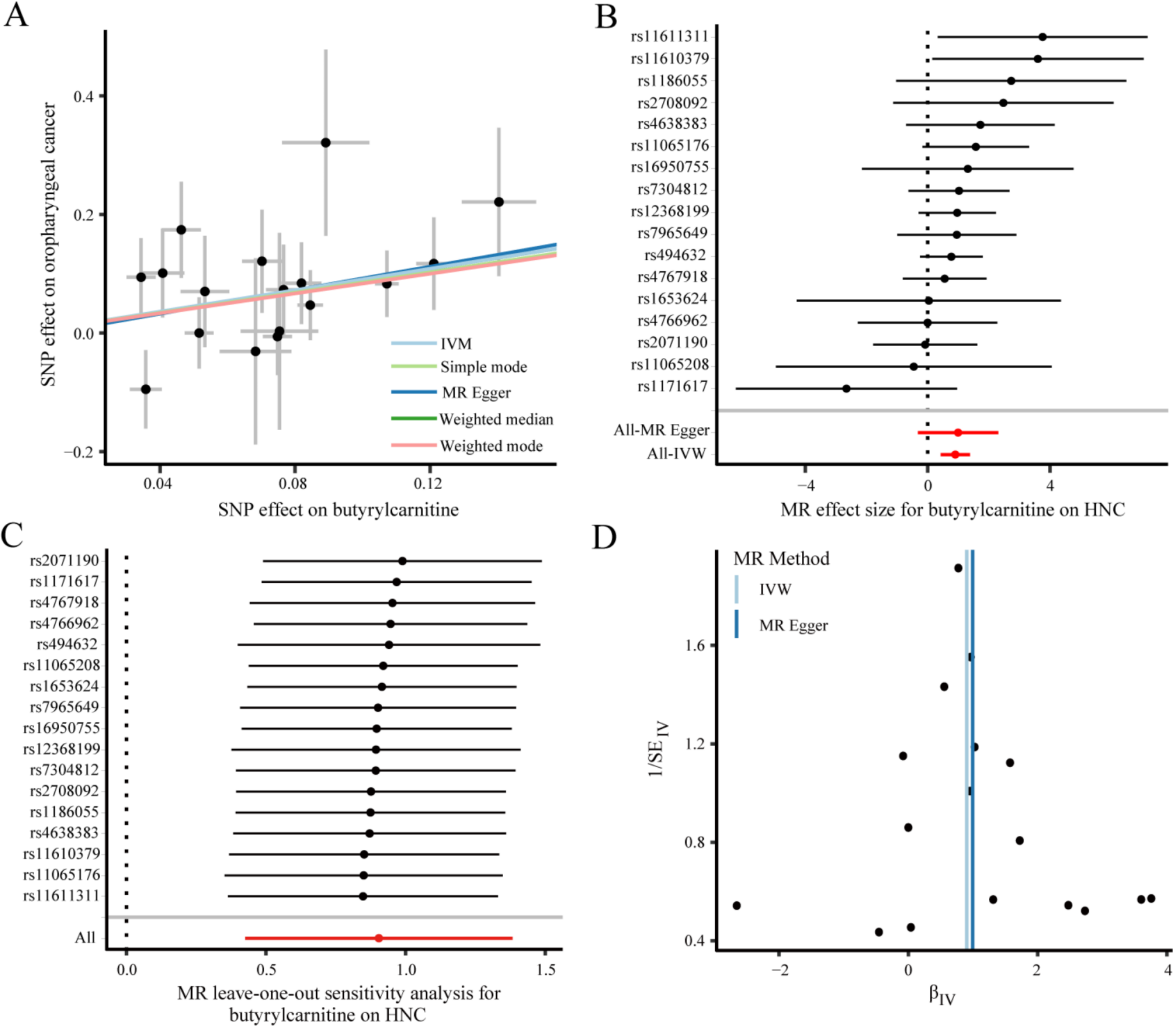
MR results of butyrylcarnitine and HNC, as well as reliability and stability analyses. (**A**) Scatter plot of genetic correlations of butyrylcarnitine and HNC using different MR methods. The slopes of line represent the causal effect of each method, respectively; (**B**) Forest plot of the causal effects of butyrylcarnitine associated SNPs on HNC. The red and black dot/bar indicate the causal estimate of butyrylcarnitine level on risk of patients with HNC; (**C**) Leave-one-out analysis for the impact of individual SNP on the association between butyrylcarnitine and the risk of HNC; (**D**) The funnel plot of the effect size for each SNP on butyrylcarnitine and the risk of HNC. HNC, head and neck cancer; IVW, inverse-variance-weighted method; MR, Mendelian randomization; SE, standard error; SNP, single nucleotide polymorphism

Our MR analyses identified causal relationships between blood metabolites and HNC, among which, only butylcarnitine (supported by 17 variants with suggestive associations) showed a statistically significant causal relationship (β = 0.904, *P* < 0.001), suggesting its potential role as a causal mediator in HNC pathogenesis (Table 1). Genetically predicted higher levels of butyrylcarnitine were positively associated with an increased risk of HNC (OR: 2.470, 95% CI: 1.530–3.987). The weighted median results supported the potential causal role of butyrylcarnitine in HNC (OR: 2.467, 95% CI: 1.287–4.728, *P* = 0.007). The directions remained consistent in the complementary analysis using genetic variants associated with butyrylcarnitine (Fig. 2A, MR Egger analysis: β = 0.995, *P* = 0.158; simple mode analysis: β = 0.854, *P* = 0.078; weighted mode analysis: β = 0.833, *P* = 0.654). Forest plots illustrating the association between butyrylcarnitine and HNC risk for the 17 SNP instruments are presented in Fig. 2B. The MR results for the remaining 23 blood metabolites are detailed in Supplementary Table 4.

**Table 1:** Nominal associations of genetically predicted butyrylcarnitine with the risk of HNC based on MR method

Outcomes	Methods	β	SE	OR (95%CI) ^a^	*P* value		Heterogeneity			Pleiotropy			*P*_steiger_
							Q ^b^	*P* value		Intercept	*P* value		
HNC cohort from GAME-ON	IVM ^c^	0.904	0.244	2.470 (1.530–3.987)	**0.000**		14.161	0.587		-0.007	0.886		0.000
	MR Egger	0.995	0.670	2.704 (0.727–10.059)	0.158								
	Simple mode	0.854	0.454	2.348 (0.965–5.713)	0.078								
	Weighted median	0.903	0.332	2.467 (1.287–4.728)	**0.007**								
	Weighted mode	0.833	0.340	2.300 (1.180–4.482)	**0.026**								

β, a ratio of changes in standard deviations; CI, confidence intervals; GAME-ON, the Genetic Associations and Mechanisms in Oncology network; IVM, inverse-variance-weighted method; HNC, head and neck cancer; MR, Mendelian randomisation; OR, odds ratio; SE, standard error

Bold represents *P* < 0.05

^a^ The estimates correspond to a standard deviation increase in butyrylcarnitine

^b^ Cochran’s Q test for heterogeneity

^c^ The estimates were derived from a fixed-effects model due to the no presence of heterogeneity based on Cochran’s Q test

### Evaluation of reliability and stability of the MR results

To ascertain the robustness of the IVW analysis outcomes, sensitivity analyses were performed. Cochran’s Q test indicated no significant heterogeneity (Q = 14.161, *P* = 0.587), supporting the reliability and consistency of the findings. MR-Egger regression method also indicated the absence of directional pleiotropy (intercept = -0.007, *P* = 0.886). The “leave-one-out” analysis demonstrated that no single SNP significantly influenced the overall effect of butyrylcarnitine on HNC risk, underscoring the reliability and stability of the results (Fig. 2C). The funnel plot exhibited symmetry, suggesting no violations in the estimates and further confirming the absence of directional pleiotropy or heterogeneity (Fig. 2D). Moreover, the MR Steiger test demonstrated no evidence of reverse causality (P_steiger_ < 0.01).

### Identification of risk SNPs

We further hypothesize that specific SNPs play a critical role in mediating the causal relationship between butyrylcarnitine and the pathogenesis of HNC. Among the 17 SNPs comprising the IVs for butyrylcarnitine, 16 are located on chromosome 12 (Supplementary Fig. 2). Notably, rs494632 exhibited an exceptionally strong association with butyrylcarnitine (*P* = 1 × 10^− 200^) (Table 2). Additionally, the SNPs rs11610379 (*P* = 0.041) and rs11611311 (*P* = 0.031) showed significant association signals with HNC. These SNPs may serve as valuable sources of information, offering potential insights into diagnostic or therapeutic targets for HNC.

**Table 2:** Annotation of the 17 risk SNPs associated with butyrylcarnitine with a causal relationship to HNC

SNP	Pos (hg19)	EA/OA	Effect on butyrylcarnitine			Effect on HNC			eQTL	Motifs changed	Gene	Annotation	Role
			β	*P* value		β	*P* value						
rs7965649	chr12:121084678	T/C	-0.077	7.23E-50		-0.073	0.341		Yes	Yes	*CABP1*	intronic	protein coding
rs4766962	chr12:120863235	T/A	-0.052	1.70E-33		0.000	0.998		Yes	No	*COX6A1*	intergenic	lncRNA
rs2071190	chr12:121431272	A/T	-0.075	8.44E-68		0.006	0.929		Yes	Yes	*HNF1A*	intronic	protein coding
rs11065208	chr12:121118172	A/G	-0.068	1.17E-10		0.031	0.845		No	Yes	*MLEC*	intergenic	protein coding
rs12368199	chr12:121472167	A/G	0.121	1.67E-121		0.117	0.133		Yes	Yes	*OASL*	intronic	protein coding
rs16950755	chr12:121488876	C/G	-0.053	1.66E-13		-0.070	0.456		Yes	Yes	*OASL*	intergenic	protein coding
rs11610379	chr12:121654720	T/C	0.089	5.09E-12		0.321	0.041		No	Yes	*P2RX4*	intronic	protein coding
rs1186055	chr12:121600529	A/C	-0.034	1.30E-15		-0.094	0.152		Yes	No	*P2RX7*	intronic	protein coding
rs1653624	chr12:121622520	T/A	0.075	4.55E-11		0.003	0.985		No	Yes	*P2RX7*	exonic	protein coding
rs7304812	chr12:121040703	A/T	0.082	2.99E-43		0.084	0.225		Yes	Yes	*POP5*	intergenic	pseudogene
rs11611311	chr12:120677587	T/A	-0.046	1.65E-15		-0.174	0.031		No	Yes	*PXN*	intronic	protein coding
rs4638383	chr12:121190302	C/G	-0.070	5.54E-33		-0.121	0.164		Yes	No	*RP11-173P15.7*	lncRNA_intronic	antisense: non-coding
rs494632	chr12:121189116	T/C	0.107	1.00E-200		0.083	0.140		Yes	Yes	*RP11-173P15.7*	lncRNA_intronic	antisense: non-coding
rs11065176	chr12:121064364	T/C	0.140	2.42E-37		0.221	0.077		No	Yes	*RP11-728G15.1*	intergenic	pseudogene
rs4767918	chr12:121053044	C/T	-0.085	2.01E-109		-0.047	0.424		Yes	No	*RP11-728G15.1*	intergenic	pseudogene
rs1171617	chr10:61467182	T/G	0.036	1.61E-14		-0.095	0.148		Yes	Yes	*SLC16A9*	intronic	protein coding
rs2708092	chr12:121525886	A/G	0.041	1.53E-10		0.101	0.180		Yes	No	*SNORA70*	intergenic	pseudogene

β, a ratio of changes in standard deviations; EA, effect allele; eQTL, expression quantitative trait locus; HNC, head and neck cancer; OA, other allele; Pos, position based on GRCh37/hg19; SNP, single nucleotide polymorphism

### Butyrylcarnitine promotes HN30 cells proliferation, invasion and migration *in vitro*

**Fig. 3:**
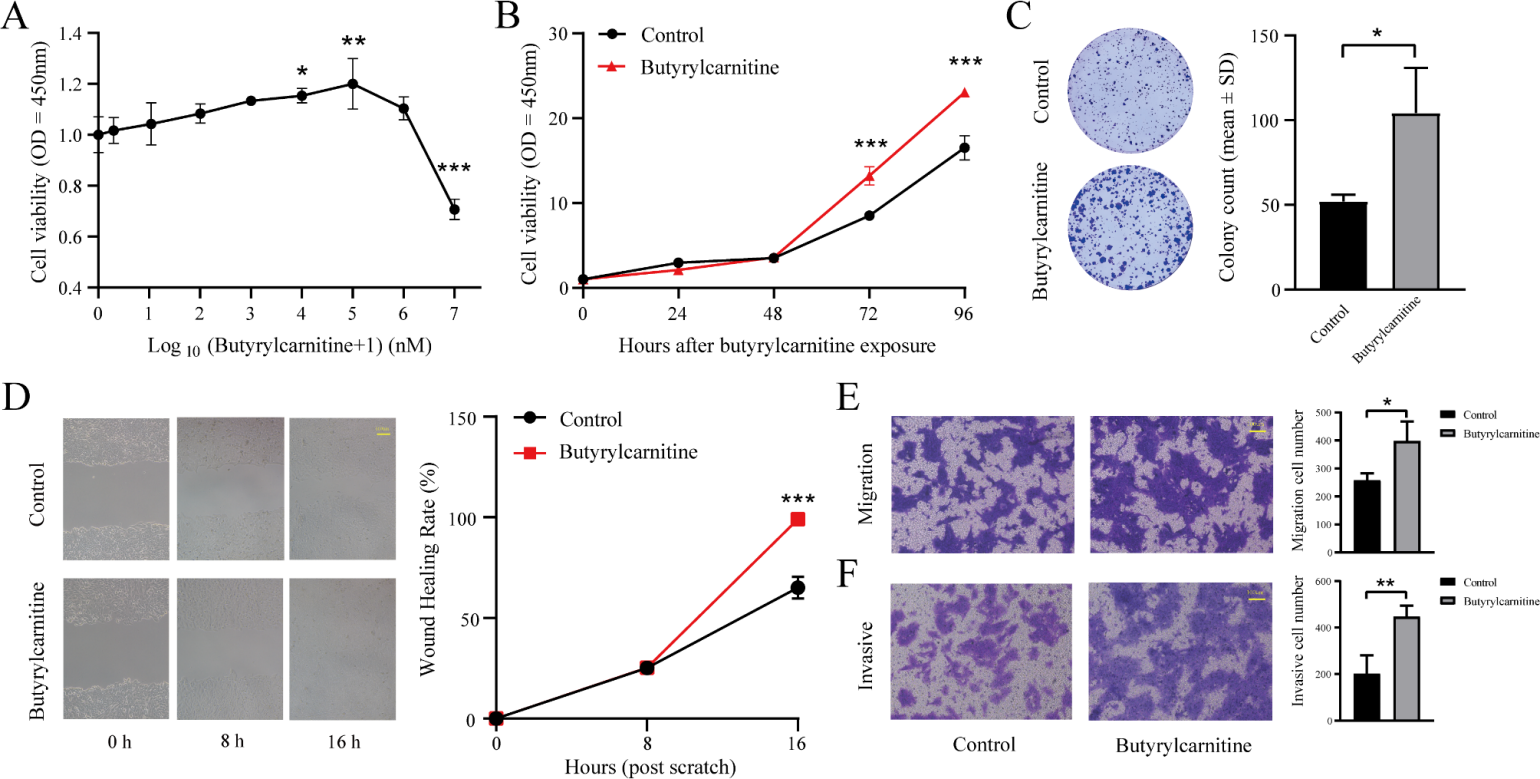
Butyrylcarnitine promotes proliferation, invasion and migration of HN30 cells. (**A**) Dose-response curve depicting the effects of varying concentrations of butyrylcarnitine (0, 1 nM, 10 nM, 100 nM, 1 µM, 10 µM, 100 µM, 1 mM, and 10 mM) on HN30 cell viability after 72 h of treatment, *P*-value is relative to the control group; (**B**) Time-dependent response curve showing the effects of 100 µM butyrylcarnitine treatment on HN30 cell viability at 0, 24, 48, 72, and 96 h; (**C**) Colony formation assay after butyrylcarnitine treatment on HN30 cells, and quantification analysis results; (**D**) Wound-healing assay after butyrylcarnitine treatment on HN30 cells at 0, 8, and 16 h post-treatment, and quantification analysis results; (**E**) Transwell migration assays after butyrylcarnitine treatment on HN30 cells, and quantification analysis results. (**F**) Transwell invasion assays after butyrylcarnitine treatment on HN30 cells, and quantification analysis results. The data are presented as the mean ± standard deviation of triplicate independent experiments. Scale bar: 100 μm. *P < 0.05; **P < 0.01; ***P < 0.001

In light of the causal relationship between butyrylcarnitine and HNC, we further investigated the effects of butyrylcarnitine on the biological behavior of HNC cells. HN30 cells were treated with varying concentrations of butyrylcarnitine, and cell viability was assessed using CCK-8 assays. The results revealed a dose-dependent increase in cell proliferation, with a significant enhancement observed at concentrations up to 100 µM (*P* < 0.01). However, higher concentrations (10 mM) led to a reduction in cell viability (*P* < 0.001, Fig. 3A). Based on these findings, 100 µM was selected as the optimal concentration for subsequent experiments. At both 72 and 96 h post-treatment, the growth rate of butyrylcarnitine-treated cells was significantly higher than that of untreated controls (Ps < 0.001, Fig. 3B). Colony formation assays further demonstrated that butyrylcarnitine significantly promoted cell proliferation (*P* < 0.05, Fig. 3C). Wound-healing assays revealed that butyrylcarnitine enhanced the ability of HN30 cells to close the wound gap at both 8 and 16 h post-treatment compared to controls (*P* < 0.001, Fig. 3D). Similarly, the migratory capacity of treated cells was significantly increased (*P* < 0.05, Fig. 3E). The Transwell invasion assay, conducted in the presence of Matrigel, showed a comparable enhancement in invasive potential following butyrylcarnitine treatment (*P* < 0.01, Fig. 3F). Collectively, these results suggest that butyrylcarnitine functions as a potent modulator of cell proliferation, migration, and invasion in HNC cells, potentially contributing to the progression of HNC.

## Discussion

This study explored the potential causal relationship between metabolites and susceptibility to HNC using two-sample MR analysis. Butyrylcarnitine was identified as a potential causal mediator in HNC pathogenesis among 258 known blood metabolites. Specifically, genetically predicted elevated levels of butyrylcarnitine were associated with an increased risk of HNC. In addition, in functional assays, we demonstrated that butyrylcarnitine exposure significantly enhanced the proliferation, invasion, and migration of HN30 cells.

Tobacco and alcohol usage, along with HPV infection have been recognized as major contributors for the development of HNC, with additional risk factors encompass genetic predisposition, exposure to toxins, dietary patterns, and environmental determinants [38]. Despite declines in tobacco- and alcohol-related HNC cases and advances in understanding HPV’s role, prevention and therapeutic interventions remain inadequate, and the biological mechanisms underlying HNC etiology are not yet well elucidated [4]. Identifying modifiable risk factors for HNC and exploring their contribution to HNC risk is highly meaningful, which may facilitate to identify individuals at risk, and provide prevention strategies. The era of personalized medicine, initiated by the completion of the human genome project, aims to leverage genetic information to develop biomarker panels for individualized cancer risk assessment and targeted therapy [2]. Recent advances have identified promising biomarkers, such as CNIH4, linked to cancer stemness and immune modulation, and AURKA, associated with cuproptosis and ferroptosis, both implicated in HNC progression [39–41]. Additionally, PANoptosis-related biomarkers, metabolism-related genes, ferroptosis-associated signature, have demonstrated prognostic value and therapeutic potential in HNC [42–44]. These findings highlight the intricate biology of HNC and reveal novel targets for precision medicine.

It has shown that metabolomics provides comprehensive measurements of metabolites in biological systems which may be associated with the occurrence and development of cancer [45]. Recently, multiple GWASs have made significant strides in identifying genetic determinants underlying human metabolome [9, 15, 16, 19]. It enables extensive cross-trait analyses at a genome-wide scale to characterize shared and distinct genetic influences across traits, facilitating the exploration of potential biological mechanisms associated with epidemiology. In our study, we identified a significant association between elevated levels of butyrylcarnitine and an increased risk of HNC through MR analysis. Furthermore, exogenous butyrylcarnitine was found to enhance the proliferation and progression of HNC cells in vitro. Acylcarnitines are ester compounds formed by the conjugation of fatty acids (acyl groups) and the amino acid L-carnitine, which plays a critical role in β-oxidation and tricarboxylic acid (TCA) cycle activity [46]. Butyrylcarnitine, a 4-carbon acylcarnitine, is synthesized through the conjugation of butyryl-CoA and carnitine [47]. Butyrylcarnitine serves as an intermediate in the breakdown of short-chain fatty acids, facilitating the generation of acetyl-CoA, which enters the TCA cycle to produce ATP and metabolic precursors for biosynthetic pathways. Elevated concentrations of acylcarnitine are hypothesized to correlate with increased mitochondrial fatty acid oxidation (FAO) rates, and abnormal FAO activation has been shown to drive the malignant progression of various cancers [48]. Increased FAO has been shown to promote cancer cell survival under metabolic stress, such as hypoxia or nutrient deprivation, by sustaining ATP production and redox balance [49]. Additionally, FAO appears to play a critical role in maintaining cancer stemness and regulate cell cycle progression and apoptosis, contributing to the aggressive progression of malignancies [48, 50]. This intricate metabolic pathway may underlie the observed positive correlation between butyrylcarnitine levels and the development of HNC.

Despite the limited epidemiological data on the role of butyrylcarnitine in HNC, previous studies have shown that elevated circulating levels of butyrylcarnitine are associated with increased risk and progression in breast cancer, while higher levels of alonylcarnitine, decenoylcarnitine, and decadienoylcarnitine were identified as protective factors [51]. However, these studies were unable to establish causal relationships due to their case-control design. In contrast, Smith-Byrne et al. demonstrated an inverse association between elevated circulating isovalerylcarnitine and lung cancer risk using an approach that integrates genetic and traditional epidemiology [16]. Moreover, a comprehensive investigation encompassing multiple cancer types, including breast, colorectal, endometrial, gallbladder, kidney, prostate, and hepatocellular carcinoma, found no significant association between butyrylcarnitine and cancer risk in cancer-specific univariate analyses, while noteworthy findings emerged from univariate pooled and data-shared lasso analyses, warranting further exploration [9]. In addition, Hatae et al. suggested that butyrylcarnitine is linked to antitumor immune responses to PD-1 blockade therapy, potentially due to FAO-mediated T cell dysfunction [52]. These findings underscore the complex interplay between metabolic processes and cancer biology, suggesting that targeting FAO may hold promise as a therapeutic strategy for inhibiting cancer growth and progression under metabolically-stressed conditions. Our study establishes a causal link between blood metabolites and HNC using MR, identifying butyrylcarnitine as a key player. Unlike previous observational studies, which are susceptible to confounding and reverse causality, our MR approach strengthens the evidence for a causal relationship and provide potential evidence of metabolic dysregulation within the framework of HNC. This metabolic alteration could guide therapeutic interventions by highlighting pathways that may be targeted to disrupt tumor metabolism. For instance, modulating FAO or its downstream effects could enhance the efficacy of existing therapies, such as immune checkpoint inhibitors, by reversing T cell dysfunction [52]. Notably, butyric acid, the precursor of butyrylcarnitine, is a short-chain fatty acid primarily derived from dietary fiber breakdown [53]. These endogenous factors are intricately linked to dietary patterns [54]. This connection suggests that dietary interventions, such as adjusting fiber intake, could modulate butyrylcarnitine levels and potentially influence HNC risk. Hence, dietary modifications represent a promising therapeutic avenue for managing HNC.

Notwithstanding the available evidence, there still exists a knowledge gap in understanding the precise biological mechanism linking butyrylcarnitine to the pathophysiology of HNC. Given the inherent relationship between metabolites, genetic makeup, and observable characteristics, numerous SNPs with significant correlations to plasma metabolites have been identified by researchers [15]. These genetic variants are situated proximal to or located at genes, and their contributions to determining metabolite levels have been well described in previous studies, which enabling us to employ SNPs near genes with influence on butyrylcarnitine levels to provide novel insights into the underlying biological mechanisms on HNC [16].

Several limitations should be considered when interpreting our findings. First, the genetic variants analyzed in this study were derived from a single cohort, future studies should aim to replicate these results in independent, diverse populations to strengthen the robustness and generalizability of the findings. Second, while MR provides robust genetic evidence for a causal relationship, residual confounding from unmeasured or incompletely controlled factors cannot be entirely ruled out. For instance, lifestyle factors such as diet, physical activity, and environmental exposures, as well as genetic factors like polygenic risk scores or gene-environment interactions, may influence the observed association between butyrylcarnitine and HNC risk. Additionally, caution must be exercised when interpreting the role of SNPs, as their functional relevance and potential pleiotropic effects require further investigation [10]. Lastly, while our in vitro experiments preliminarily demonstrated the effects of butyrylcarnitine on HNC cells, this does not exclude the possibility of other metabolites within the butyrylcarnitine pathway contributing to HNC pathogenesis. Further research is needed to comprehensively explore the constituents of this pathway and elucidate the intricate mechanisms underlying the association between SNPs and HNC, both in vitro and in vivo.

## Conclusions

In summary, our results demonstrate that butyrylcarnitine exerts a positive causal effect on the development of HNC. Butyrylcarnitine exhibits carcinogenic activity against HNC cell line. These findings provide novel insights into the metabolic and genetic factors driving HNC development and highlight potential therapeutic targets for the treatment of HNC.

## Electronic supplementary material

Below is the link to the electronic supplementary material.

Supplementary Material 1

Supplementary Material 2

## Data Availability

Summary-level data analysed during the current study are available from the web Open GWAS (https://gwas.mrcieu.ac.uk/); Potential genetic correlation between SNPs and outcome were searched in the web tool PhenoScanner (version 2, http://www.phenoscanner.medschl.cam.ac.uk/); Functional annotation was used online tools FUMA GWAS (https://fuma.ctglab.nl/); Statistical analysis software R software (v4.0.4) was downloaded from https://www.r-project.org/.

## Abbreviations

CCK-8: Cell counting kit-8
FAO: Fatty acid β-oxidation
FUMA GWAS: Functional mapping and annotation of genome-wide association studies
GAME-ON: Genetic associations and mechanisms in oncology
GWAS: Genome-wide association study
HNC: Head and neck cancer
HPV: Human papillomavirus
ICD: International classification of diseases
IEU: Integrative epidemiology unit
IVs: Instrumental variables
IVW: Inverse-variance-weighted method
LD: Linkage disequilibrium
MAFs: Minor allele frequencies
MR: Mendelian randomization
SNPs: Single nucleotide polymorphisms
TCA: Tricarboxylic acid
