# Supplementary material for: Causal association between blood metabolites and head and neck cancer: butyrylcarnitine identified as an associated trait for cancer risk and progression
Source: Hereditas. 2025 Mar 14;162:36. doi: 10.1186/s41065-025-00408-5 (PMC11907814; doi:10.1186/s41065-025-00408-5)
Supplement: Supplementary file 1 — Supplementary Material 1 [file 41065_2025_408_MOESM1_ESM.docx]

| **Supplementary Table 1** Detailed information on the GWAS^*^ applied in the present study | | | | | | | | |
| --- | --- | --- | --- | --- | --- | --- | --- | --- |
| **Outcomes/Exposures** | **Cohorts or datasets** | **[GWAS ID](https://gwas.mrcieu.ac.uk/datasets/?trait__icontains=head+and+neck+cancer&sort=-gwas_id)** | **Year** | **[Consortium](https://gwas.mrcieu.ac.uk/datasets/?trait__icontains=head+and+neck+cancer&sort=consortium)** | **[Sample size (Cases/Controls)](https://gwas.mrcieu.ac.uk/datasets/?trait__icontains=head+and+neck+cancer&sort=sample_size)** | **[Number of SNPs](https://gwas.mrcieu.ac.uk/datasets/?trait__icontains=head+and+neck+cancer&sort=nsnp)** | **Population** | **Data source** |
| HNC cohort | GAME-ON | ieu-b-96 | 2016 | Oncoarray oral cavity and oropharyngeal cancer | 1090/2928 | 7508444 | European | PMID: 27749845 |
| Human blood metabolites | TwinsUK2012 ^a^ TwinsUK2014 ^b^ KORA2011 ^c^ | met-a | 2014 | NA | 7824 | NA | European | PMID: 24816252 |
| GAME-ON, the Genetic Associations and Mechanisms in Oncology network; GWAS, Genome-Wide Association Studies; HNC, head and neck cancer; SNP, single nucleotide polymorphism. ^a^ the study from Krumsiek J et al.[16] ^b^ the study from Shin SY et al.[11] ^c^ the study from Suhre K et al. [17] ^*^ from the web Open GWAS (https://gwas.mrcieu.ac.uk/) | | | | | | | | |

| **Supplementary Table 2** 316 independent SNPs associated with blood metabolites | | | | | | | | | |
| --- | --- | --- | --- | --- | --- | --- | --- | --- | --- |
| **SNP** | **EA** | **OA** | **P value** | **β** | **SE** | **EAF** | **F ^a^** | **R^2^ (%) ^b^** | **Exposure** |
| rs2657879 | G | A | 6.15E-18 | -0.015 | 0.002 | 0.182 | 77.855 | 0.296 | Glutamine |
| rs13122250 | T | C | 8.95E-12 | 0.006 | 0.001 | 0.554 | 47.457 | 0.300 | Tryptophan |
| rs1016522 | A | G | 1.59E-10 | 0.006 | 0.001 | 0.580 | 41.531 | 0.259 | Tryptophan |
| rs6901004 | G | C | 1.08E-11 | 0.006 | 0.001 | 0.426 | 45.938 | 0.287 | Tryptophan |
| rs16868246 | G | C | 1.78E-14 | -0.010 | 0.001 | 0.789 | 55.675 | 0.237 | Histidine |
| rs1440581 | C | T | 1.44E-19 | 0.008 | 0.001 | 0.541 | 81.000 | 0.514 | Leucine |
| rs1718306 | C | T | 7.34E-11 | -0.008 | 0.001 | 0.598 | 46.488 | 0.286 | Phenylalanine |
| rs1260326 | C | T | 1.30E-77 | 0.044 | 0.002 | 0.591 | 334.585 | 2.067 | Mannose |
| rs7583698 | T | C | 8.55E-12 | 0.034 | 0.005 | 0.048 | 47.582 | 0.056 | Mannose |
| rs6547811 | A | T | 7.10E-12 | 0.022 | 0.003 | 0.158 | 48.129 | 0.164 | Mannose |
| rs2141371 | A | G | 3.27E-16 | -0.020 | 0.002 | 0.682 | 68.063 | 0.377 | Mannose |
| rs131794 | C | A | 4.37E-16 | -0.015 | 0.002 | 0.793 | 65.790 | 0.276 | Uridine |
| rs412334 | T | C | 2.24E-13 | 0.022 | 0.003 | 0.157 | 53.778 | 0.182 | Arachidonate (20:4n6) |
| rs174548 | G | C | 1.43E-84 | -0.049 | 0.003 | 0.300 | 381.030 | 2.043 | Arachidonate (20:4n6) |
| rs174602 | C | T | 2.42E-18 | -0.035 | 0.004 | 0.130 | 77.881 | 0.225 | Arachidonate (20:4n6) |
| rs603424 | A | G | 1.56E-14 | 0.029 | 0.004 | 0.187 | 59.047 | 0.229 | Margarate (17:0) |
| rs9450282 | G | A | 2.74E-11 | 0.072 | 0.011 | 0.637 | 44.075 | 0.260 | Inosine |
| rs494562 | G | A | 1.34E-12 | 0.118 | 0.017 | 0.101 | 50.096 | 0.116 | Inosine |
| rs9400467 | T | C | 6.54E-14 | -0.012 | 0.002 | 0.706 | 57.191 | 0.304 | Tyrosine |
| rs2863979 | A | G | 1.44E-17 | 0.014 | 0.002 | 0.724 | 70.758 | 0.361 | Lysine |
| rs603424 | A | G | 6.06E-21 | 0.023 | 0.002 | 0.184 | 87.891 | 0.337 | Stearate (18:0) |
| rs12550729 | T | C | 5.97E-110 | 0.064 | 0.003 | 0.923 | 482.484 | 0.873 | 5-oxoproline |
| rs3936211 | T | C | 1.34E-14 | -0.012 | 0.002 | 0.424 | 62.938 | 0.393 | 5-oxoproline |
| rs1395 | A | G | 8.14E-11 | 0.017 | 0.003 | 0.703 | 40.582 | 0.216 | Pantothenate |
| rs9657488 | C | T | 3.53E-12 | -0.012 | 0.002 | 0.625 | 47.457 | 0.284 | 4-acetamidobutanoate |
| rs13271577 | T | C | 2.56E-17 | 0.022 | 0.003 | 0.138 | 70.948 | 0.216 | 4-acetamidobutanoate |
| rs1041983 | T | C | 2.14E-19 | -0.016 | 0.002 | 0.323 | 80.003 | 0.447 | 4-acetamidobutanoate |
| rs721399 | T | C | 3.95E-58 | -0.029 | 0.002 | 0.718 | 257.781 | 1.335 | 4-acetamidobutanoate |
| rs835154 | A | G | 1.15E-10 | -0.011 | 0.002 | 0.575 | 40.111 | 0.251 | Citrate |
| rs170149 | A | G | 1.42E-13 | 0.026 | 0.004 | 0.074 | 53.499 | 0.094 | Citrate |
| rs938554 | G | C | 5.85E-93 | 0.035 | 0.002 | 0.774 | 419.045 | 1.871 | Urate |
| rs11731100 | T | A | 9.97E-13 | 0.012 | 0.002 | 0.650 | 52.563 | 0.306 | Urate |
| rs11737601 | G | A | 1.34E-10 | -0.010 | 0.002 | 0.315 | 42.250 | 0.233 | Urate |
| rs4697910 | A | G | 8.72E-14 | -0.012 | 0.002 | 0.287 | 53.204 | 0.278 | Urate |
| rs10939663 | G | T | 5.57E-22 | 0.016 | 0.002 | 0.272 | 89.692 | 0.454 | Urate |
| rs1440581 | C | T | 2.56E-12 | 0.008 | 0.001 | 0.542 | 45.563 | 0.289 | Valine |
| rs5746636 | T | G | 3.94E-19 | -0.032 | 0.004 | 0.198 | 78.519 | 0.318 | Proline |
| rs5747934 | T | C | 2.00E-44 | 0.072 | 0.005 | 0.047 | 196.549 | 0.224 | Proline |
| rs9605907 | G | A | 9.70E-19 | 0.023 | 0.003 | 0.838 | 77.575 | 0.270 | Proline |
| rs2518802 | C | A | 4.15E-59 | 0.053 | 0.003 | 0.097 | 261.851 | 0.586 | Proline |
| rs1935815 | A | T | 1.55E-10 | 0.011 | 0.002 | 0.351 | 42.633 | 0.248 | Citrulline |
| rs6717546 | G | A | 3.93E-28 | 0.040 | 0.004 | 0.635 | 120.390 | 0.713 | Biliverdin |
| rs28900371 | A | G | 7.65E-19 | 0.089 | 0.010 | 0.035 | 79.210 | 0.069 | Biliverdin |
| rs7608713 | A | G | 7.35E-21 | -0.042 | 0.004 | 0.240 | 88.959 | 0.415 | Biliverdin |
| rs887829 | T | C | 2.50E-168 | 0.113 | 0.004 | 0.341 | 760.952 | 4.372 | Biliverdin |
| rs2219067 | G | C | 1.34E-19 | -0.040 | 0.004 | 0.239 | 81.410 | 0.378 | Biliverdin |
| rs17864661 | T | C | 1.56E-14 | 0.054 | 0.007 | 0.089 | 59.510 | 0.123 | Biliverdin |
| rs838705 | A | G | 1.23E-13 | 0.027 | 0.004 | 0.613 | 54.596 | 0.331 | Biliverdin |
| rs2657879 | G | A | 5.85E-19 | -0.024 | 0.003 | 0.174 | 77.701 | 0.285 | Gamma-glutamylglutamine |
| rs6459467 | A | G | 1.93E-16 | 0.020 | 0.003 | 0.389 | 65.286 | 0.397 | Hypoxanthine |
| rs715 | C | T | 2.08E-19 | -0.022 | 0.003 | 0.287 | 78.146 | 0.409 | Betaine |
| rs16876394 | C | T | 1.49E-19 | -0.030 | 0.003 | 0.101 | 79.945 | 0.185 | Betaine |
| rs185077 | C | T | 1.54E-16 | 0.019 | 0.002 | 0.501 | 70.414 | 0.450 | Betaine |
| rs2851391 | C | T | 1.15E-11 | 0.012 | 0.002 | 0.542 | 45.188 | 0.287 | Betaine |
| rs3184504 | C | T | 6.05E-18 | -0.015 | 0.002 | 0.515 | 76.820 | 0.490 | Kynurenine |
| rs8051149 | A | G | 9.07E-26 | 0.026 | 0.003 | 0.213 | 106.502 | 0.456 | Kynurenine |
| rs750950 | C | A | 2.34E-16 | -0.014 | 0.002 | 0.356 | 64.000 | 0.375 | Kynurenine |
| rs1466788 | G | A | 3.05E-16 | 0.007 | 0.001 | 0.593 | 67.605 | 0.417 | Carnitine |
| rs9842133 | C | T | 4.20E-12 | -0.006 | 0.001 | 0.335 | 50.568 | 0.288 | Carnitine |
| rs419291 | C | T | 3.10E-18 | -0.008 | 0.001 | 0.633 | 77.049 | 0.457 | Carnitine |
| rs7737937 | A | G | 4.91E-11 | -0.011 | 0.002 | 0.151 | 41.111 | 0.135 | Carnitine |
| rs6479648 | A | G | 8.40E-22 | -0.009 | 0.001 | 0.420 | 93.444 | 0.582 | Carnitine |
| rs12356193 | G | A | 3.69E-63 | -0.027 | 0.002 | 0.164 | 293.266 | 1.027 | Carnitine |
| rs1171618 | C | T | 1.58E-59 | 0.015 | 0.001 | 0.381 | 281.494 | 1.697 | Carnitine |
| rs7098081 | A | G | 3.10E-13 | 0.018 | 0.003 | 0.047 | 54.170 | 0.062 | Carnitine |
| rs1318987 | A | C | 1.81E-54 | -0.092 | 0.006 | 0.157 | 242.620 | 0.820 | N-acetylornithine |
| rs7573275 | A | G | 1.00E-200 | -0.151 | 0.004 | 0.274 | 1294.286 | 6.580 | N-acetylornithine |
| rs1881244 | A | G | 2.91E-112 | -0.191 | 0.009 | 0.063 | 503.342 | 0.760 | N-acetylornithine |
| rs2043099 | A | G | 1.09E-32 | -0.051 | 0.004 | 0.271 | 142.886 | 0.722 | N-acetylornithine |
| rs17348756 | T | C | 1.87E-12 | 0.048 | 0.007 | 0.108 | 50.262 | 0.124 | N-acetylornithine |
| rs17349853 | G | T | 1.82E-16 | 0.074 | 0.009 | 0.070 | 67.240 | 0.112 | N-acetylornithine |
| rs1440581 | C | T | 1.21E-16 | 0.014 | 0.002 | 0.541 | 67.820 | 0.430 | 3-methyl-2-oxovalerate |
| rs6497490 | T | G | 1.91E-10 | -0.048 | 0.008 | 0.882 | 40.450 | 0.108 | 3-phenylpropionate (hydrocinnamate) |
| rs11647589 | G | A | 2.85E-11 | -0.030 | 0.005 | 0.285 | 45.338 | 0.236 | 3-phenylpropionate (hydrocinnamate) |
| rs1030420 | T | C | 1.02E-11 | -0.022 | 0.003 | 0.188 | 47.696 | 0.186 | X-04499--3,4-dihydroxybutyrate |
| rs15676 | A | G | 1.33E-12 | 0.019 | 0.003 | 0.712 | 50.043 | 0.262 | Indolelactate |
| rs174556 | T | C | 1.97E-22 | -0.036 | 0.004 | 0.299 | 92.576 | 0.496 | Eicosapentaenoate (EPA; 20:5n3) |
| rs4788439 | T | C | 6.59E-13 | -0.027 | 0.004 | 0.077 | 50.859 | 0.093 | Myo-inositol |
| rs4808136 | A | G | 5.42E-14 | 0.013 | 0.002 | 0.339 | 53.778 | 0.308 | Myo-inositol |
| rs7570971 | A | C | 7.86E-45 | -0.037 | 0.003 | 0.326 | 190.849 | 1.071 | 1,5-anhydroglucitol (1,5-AG) |
| rs3800993 | T | C | 1.53E-15 | 0.029 | 0.004 | 0.170 | 63.556 | 0.229 | 1,5-anhydroglucitol (1,5-AG) |
| rs1795963 | G | A | 1.27E-15 | 0.021 | 0.003 | 0.623 | 64.000 | 0.384 | 2-hydroxyisobutyrate |
| rs493519 | C | T | 1.32E-18 | 0.036 | 0.004 | 0.875 | 76.243 | 0.213 | 2-hydroxyisobutyrate |
| rs1657179 | T | C | 9.38E-15 | -0.021 | 0.003 | 0.540 | 59.347 | 0.377 | Phenyllactate (PLA) |
| rs4784054 | A | G | 2.31E-21 | 0.035 | 0.004 | 0.106 | 88.971 | 0.216 | Phenyllactate (PLA) |
| rs8101881 | T | C | 1.43E-16 | 0.033 | 0.004 | 0.613 | 69.444 | 0.421 | Homocitrulline |
| rs4351 | A | G | 1.24E-19 | -0.046 | 0.005 | 0.475 | 82.810 | 0.528 | Aspartylphenylalanine |
| rs13278849 | A | G | 2.79E-11 | -0.013 | 0.002 | 0.730 | 42.250 | 0.213 | Levulinate (4-oxovalerate) |
| rs11825181 | A | G | 2.93E-11 | 0.076 | 0.011 | 0.077 | 44.561 | 0.081 | 1-linoleoylglycerol (1-monolinolein) |
| rs7809234 | T | A | 1.60E-10 | -0.029 | 0.005 | 0.125 | 40.111 | 0.112 | Indoleacetate |
| rs13021675 | T | C | 5.60E-16 | 0.049 | 0.006 | 0.197 | 65.340 | 0.264 | N-acetylglycine |
| rs1990797 | C | G | 1.33E-15 | -0.049 | 0.006 | 0.171 | 63.476 | 0.230 | N-acetylglycine |
| rs1367053 | C | T | 4.88E-12 | -0.031 | 0.004 | 0.546 | 48.366 | 0.307 | N-acetylglycine |
| rs715 | C | T | 6.66E-58 | 0.091 | 0.006 | 0.290 | 254.878 | 1.343 | N-acetylglycine |
| rs12468557 | T | C | 1.84E-11 | -0.029 | 0.004 | 0.373 | 44.343 | 0.265 | N-acetylglycine |
| rs6760588 | C | A | 1.13E-18 | -0.060 | 0.007 | 0.252 | 77.078 | 0.371 | Bilirubin (Z,Z) |
| rs3796092 | A | C | 4.53E-16 | 0.080 | 0.010 | 0.098 | 66.140 | 0.149 | Bilirubin (Z,Z) |
| rs12479240 | T | C | 3.11E-11 | -0.055 | 0.008 | 0.169 | 43.751 | 0.157 | Bilirubin (Z,Z) |
| rs887829 | T | C | 2.15E-152 | 0.168 | 0.006 | 0.327 | 686.604 | 3.859 | Bilirubin (Z,Z) |
| rs838718 | A | G | 3.45E-11 | 0.039 | 0.006 | 0.485 | 44.291 | 0.283 | Bilirubin (Z,Z) |
| rs28900385 | C | T | 2.85E-18 | 0.141 | 0.016 | 0.033 | 76.264 | 0.062 | Bilirubin (Z,Z) |
| rs3771342 | T | G | 6.38E-11 | -0.061 | 0.009 | 0.112 | 42.388 | 0.108 | Bilirubin (Z,Z) |
| rs6717546 | G | A | 2.02E-21 | 0.057 | 0.006 | 0.635 | 91.705 | 0.544 | Bilirubin (Z,Z) |
| rs715 | C | T | 9.63E-25 | 0.045 | 0.004 | 0.289 | 105.063 | 0.552 | Creatine |
| rs474229 | T | G | 2.92E-22 | 0.063 | 0.007 | 0.601 | 94.239 | 0.577 | Androsterone sulfate |
| rs10278040 | A | G | 8.81E-113 | -0.361 | 0.016 | 0.042 | 508.784 | 0.522 | Androsterone sulfate |
| rs7778571 | G | A | 3.50E-18 | -0.063 | 0.007 | 0.246 | 74.953 | 0.355 | Androsterone sulfate |
| rs13222543 | T | C | 6.80E-74 | -0.477 | 0.026 | 0.020 | 331.462 | 0.165 | Androsterone sulfate |
| rs6465737 | G | T | 1.55E-11 | 0.050 | 0.007 | 0.778 | 45.654 | 0.202 | Androsterone sulfate |
| rs182420 | T | C | 6.24E-12 | 0.051 | 0.007 | 0.755 | 47.498 | 0.224 | Androsterone sulfate |
| rs4625783 | C | T | 3.90E-13 | 0.019 | 0.003 | 0.573 | 51.729 | 0.323 | 3-(4-hydroxyphenyl)lactate |
| rs274567 | T | C | 2.85E-12 | 0.018 | 0.003 | 0.371 | 49.562 | 0.296 | Acetylcarnitine |
| rs1171614 | C | T | 3.38E-23 | 0.028 | 0.003 | 0.777 | 99.287 | 0.440 | Acetylcarnitine |
| rs1163251 | C | T | 7.05E-27 | -0.019 | 0.002 | 0.400 | 122.298 | 0.750 | Serine |
| rs715 | C | T | 2.69E-21 | 0.022 | 0.002 | 0.289 | 86.335 | 0.453 | Serine |
| rs4947534 | C | T | 1.96E-14 | 0.018 | 0.002 | 0.752 | 58.141 | 0.277 | Serine |
| rs17304141 | G | T | 2.46E-26 | 0.128 | 0.012 | 0.021 | 112.255 | 0.058 | Hexanoylcarnitine |
| rs721204 | A | G | 3.00E-23 | 0.050 | 0.005 | 0.112 | 98.406 | 0.249 | Hexanoylcarnitine |
| rs5745515 | T | C | 3.12E-14 | 0.043 | 0.006 | 0.094 | 58.413 | 0.127 | Hexanoylcarnitine |
| rs11161521 | C | T | 6.42E-100 | -0.070 | 0.003 | 0.302 | 453.819 | 2.444 | Hexanoylcarnitine |
| rs272869 | G | A | 1.40E-11 | -0.022 | 0.003 | 0.629 | 46.075 | 0.275 | Hexanoylcarnitine |
| rs1171615 | T | C | 4.05E-11 | 0.025 | 0.004 | 0.778 | 44.205 | 0.195 | Hexanoylcarnitine |
| rs10932349 | G | C | 7.45E-11 | -0.031 | 0.005 | 0.079 | 42.250 | 0.078 | Glycine |
| rs16845044 | C | A | 6.00E-11 | -0.021 | 0.003 | 0.168 | 43.478 | 0.155 | Glycine |
| rs13401425 | A | G | 6.88E-19 | -0.022 | 0.002 | 0.364 | 80.252 | 0.475 | Glycine |
| rs13021675 | T | C | 3.88E-27 | 0.035 | 0.003 | 0.197 | 116.910 | 0.473 | Glycine |
| rs12328639 | G | A | 8.50E-22 | -0.032 | 0.003 | 0.151 | 92.276 | 0.302 | Glycine |
| rs13011429 | C | G | 5.38E-15 | -0.029 | 0.004 | 0.129 | 62.282 | 0.179 | Glycine |
| rs715 | C | T | 1.58E-147 | 0.076 | 0.003 | 0.292 | 686.801 | 3.630 | Glycine |
| rs1990797 | C | G | 6.96E-28 | -0.036 | 0.003 | 0.169 | 116.378 | 0.418 | Glycine |
| rs2719966 | C | A | 7.31E-19 | -0.022 | 0.002 | 0.549 | 81.000 | 0.513 | Glycine |
| rs1260326 | C | T | 5.56E-14 | -0.013 | 0.002 | 0.595 | 59.381 | 0.366 | Alanine |
| rs10211524 | A | G | 5.59E-16 | 0.019 | 0.002 | 0.419 | 67.526 | 0.420 | 2-aminobutyrate |
| rs10022462 | T | C | 4.55E-11 | -0.012 | 0.002 | 0.452 | 42.250 | 0.268 | 2-aminobutyrate |
| rs4787294 | T | A | 9.64E-21 | -0.075 | 0.008 | 0.072 | 87.891 | 0.150 | Scyllo-inositol |
| rs6497490 | T | G | 6.26E-18 | -0.059 | 0.007 | 0.882 | 75.026 | 0.199 | Indolepropionate |
| rs1394678 | T | C | 1.70E-20 | -0.035 | 0.004 | 0.279 | 86.784 | 0.446 | Indolepropionate |
| rs1171617 | T | G | 1.61E-14 | 0.036 | 0.005 | 0.777 | 58.019 | 0.257 | Butyrylcarnitine |
| rs11610379 | T | C | 5.09E-12 | 0.089 | 0.013 | 0.027 | 47.706 | 0.032 | Butyrylcarnitine |
| rs16950755 | C | G | 1.66E-13 | -0.053 | 0.007 | 0.083 | 54.801 | 0.107 | Butyrylcarnitine |
| rs11065208 | A | G | 1.17E-10 | -0.068 | 0.011 | 0.034 | 41.517 | 0.034 | Butyrylcarnitine |
| rs1653624 | T | A | 4.55E-11 | 0.075 | 0.012 | 0.972 | 42.988 | 0.030 | Butyrylcarnitine |
| rs11611311 | T | A | 1.65E-15 | -0.046 | 0.006 | 0.142 | 63.724 | 0.199 | Butyrylcarnitine |
| rs494632 | T | C | 1.00E-200 | 0.107 | 0.004 | 0.479 | 938.109 | 5.984 | Butyrylcarnitine |
| rs4767918 | C | T | 2.01E-109 | -0.085 | 0.004 | 0.411 | 494.477 | 3.059 | Butyrylcarnitine |
| rs12368199 | A | G | 1.67E-121 | 0.121 | 0.005 | 0.146 | 542.352 | 1.731 | Butyrylcarnitine |
| rs4766962 | T | A | 1.70E-33 | -0.052 | 0.004 | 0.346 | 144.000 | 0.832 | Butyrylcarnitine |
| rs7304812 | A | T | 2.99E-43 | 0.082 | 0.006 | 0.221 | 192.692 | 0.847 | Butyrylcarnitine |
| rs11065176 | T | C | 2.42E-37 | 0.140 | 0.011 | 0.037 | 162.910 | 0.148 | Butyrylcarnitine |
| rs7965649 | T | C | 7.23E-50 | -0.077 | 0.005 | 0.159 | 216.996 | 0.741 | Butyrylcarnitine |
| rs11065202 | C | T | 1.00E-200 | 0.130 | 0.004 | 0.418 | 1051.381 | 6.537 | Butyrylcarnitine |
| rs2708092 | A | G | 1.53E-10 | 0.041 | 0.006 | 0.823 | 40.641 | 0.152 | Butyrylcarnitine |
| rs208294 | C | T | 1.08E-18 | -0.032 | 0.004 | 0.565 | 76.563 | 0.481 | Butyrylcarnitine |
| rs4638383 | C | G | 5.54E-33 | -0.070 | 0.006 | 0.118 | 141.570 | 0.377 | Butyrylcarnitine |
| rs2071190 | A | T | 8.44E-68 | -0.075 | 0.004 | 0.250 | 302.598 | 1.450 | Butyrylcarnitine |
| rs1186055 | A | C | 1.30E-15 | -0.034 | 0.004 | 0.294 | 64.000 | 0.340 | Butyrylcarnitine |
| rs603424 | A | G | 7.16E-16 | -0.037 | 0.005 | 0.185 | 64.000 | 0.247 | Myristoleate (14:1n5) |
| rs11761528 | T | C | 6.70E-14 | -0.068 | 0.009 | 0.074 | 56.250 | 0.098 | Dehydroisoandrosterone sulfate (DHEA-S) |
| rs296396 | C | T | 1.17E-12 | 0.046 | 0.007 | 0.835 | 50.519 | 0.178 | Dehydroisoandrosterone sulfate (DHEA-S) |
| rs7727544 | T | C | 6.61E-16 | -0.015 | 0.002 | 0.555 | 67.605 | 0.427 | Propionylcarnitine |
| rs662138 | G | C | 3.15E-14 | -0.021 | 0.003 | 0.170 | 58.211 | 0.210 | Propionylcarnitine |
| rs12356193 | G | A | 1.96E-33 | -0.033 | 0.003 | 0.164 | 147.578 | 0.517 | Propionylcarnitine |
| rs9333029 | G | A | 1.52E-61 | 0.070 | 0.004 | 0.133 | 273.824 | 0.808 | 10-undecenoate (11:1n1) |
| rs2494248 | C | T | 1.19E-14 | 0.025 | 0.003 | 0.329 | 61.524 | 0.347 | 10-undecenoate (11:1n1) |
| rs7556052 | T | A | 5.50E-24 | 0.035 | 0.003 | 0.328 | 103.561 | 0.583 | 10-undecenoate (11:1n1) |
| rs174538 | A | G | 8.90E-14 | -0.026 | 0.004 | 0.303 | 56.895 | 0.307 | Docosapentaenoate (n3 DPA; 22:5n3) |
| rs887829 | T | C | 3.76E-95 | 0.128 | 0.006 | 0.330 | 426.223 | 2.407 | Bilirubin (E,E) |
| rs7608713 | A | G | 1.28E-11 | -0.045 | 0.007 | 0.238 | 45.870 | 0.213 | Bilirubin (E,E) |
| rs28900371 | A | G | 7.07E-12 | 0.105 | 0.015 | 0.035 | 46.665 | 0.040 | Bilirubin (E,E) |
| rs6723936 | A | G | 2.41E-16 | 0.047 | 0.006 | 0.641 | 66.788 | 0.393 | Bilirubin (E,E) |
| rs174535 | C | T | 2.82E-36 | 0.044 | 0.004 | 0.332 | 158.041 | 0.896 | 1-linoleoylglycerophosphoethanolamine |
| rs7737937 | A | G | 7.89E-22 | -0.032 | 0.003 | 0.151 | 90.250 | 0.296 | 3-dehydrocarnitine |
| rs13355301 | G | C | 1.04E-10 | -0.043 | 0.007 | 0.031 | 41.661 | 0.031 | 3-dehydrocarnitine |
| rs273913 | C | T | 1.08E-25 | -0.026 | 0.003 | 0.618 | 108.994 | 0.658 | 3-dehydrocarnitine |
| rs2291428 | C | G | 8.71E-11 | -0.017 | 0.003 | 0.235 | 41.751 | 0.192 | 3-dehydrocarnitine |
| rs715 | C | T | 2.46E-16 | -0.036 | 0.004 | 0.286 | 67.315 | 0.351 | Pyroglutamine |
| rs17279437 | A | G | 1.25E-20 | 0.059 | 0.006 | 0.095 | 87.705 | 0.193 | Pyroglutamine |
| rs11613331 | A | G | 2.23E-25 | 0.037 | 0.004 | 0.553 | 107.929 | 0.682 | Pyroglutamine |
| rs6909681 | A | T | 1.21E-14 | 0.020 | 0.003 | 0.472 | 58.581 | 0.373 | X-03056--N-[3-(2-Oxopyrrolidin-1-yl)propyl]acetamide |
| rs662138 | G | C | 1.03E-22 | -0.034 | 0.004 | 0.169 | 95.481 | 0.343 | X-03056--N-[3-(2-Oxopyrrolidin-1-yl)propyl]acetamide |
| rs1005390 | G | T | 2.50E-14 | -0.026 | 0.003 | 0.200 | 58.928 | 0.241 | X-03056--N-[3-(2-Oxopyrrolidin-1-yl)propyl]acetamide |
| rs11101730 | T | A | 3.27E-13 | 0.055 | 0.008 | 0.059 | 52.945 | 0.075 | X-03056--N-[3-(2-Oxopyrrolidin-1-yl)propyl]acetamide |
| rs12602901 | C | G | 1.14E-26 | -0.037 | 0.003 | 0.172 | 117.149 | 0.426 | X-03056--N-[3-(2-Oxopyrrolidin-1-yl)propyl]acetamide |
| rs6151429 | C | T | 6.51E-18 | 0.029 | 0.003 | 0.071 | 72.250 | 0.122 | X-11423--O-sulfo-L-tyrosine |
| rs10491431 | A | C | 1.59E-11 | 0.081 | 0.012 | 0.150 | 45.226 | 0.148 | X-11445--5-alpha-pregnan-3beta,20alpha-disulfate |
| rs438798 | G | A | 1.55E-23 | -0.017 | 0.002 | 0.465 | 103.561 | 0.659 | X-11593--O-methylascorbate |
| rs4597638 | C | T | 6.06E-11 | 0.028 | 0.004 | 0.058 | 42.705 | 0.060 | X-11593--O-methylascorbate |
| rs4680 | A | G | 4.66E-178 | -0.049 | 0.002 | 0.507 | 817.287 | 5.222 | X-11593--O-methylascorbate |
| rs3804043 | A | C | 7.76E-24 | 0.018 | 0.002 | 0.291 | 97.790 | 0.516 | X-11593--O-methylascorbate |
| rs5746846 | G | C | 7.81E-46 | 0.025 | 0.002 | 0.527 | 207.699 | 1.324 | X-11593--O-methylascorbate |
| rs887200 | T | C | 8.02E-30 | -0.029 | 0.003 | 0.881 | 127.864 | 0.343 | X-11593--O-methylascorbate |
| rs7290062 | C | T | 2.24E-11 | 0.017 | 0.003 | 0.137 | 45.158 | 0.136 | X-11593--O-methylascorbate |
| rs174550 | C | T | 6.79E-24 | -0.035 | 0.003 | 0.332 | 104.160 | 0.591 | Adrenate (22:4n6) |
| rs4253236 | C | T | 1.25E-12 | 0.071 | 0.010 | 0.638 | 50.410 | 0.298 | ADSGEGDFXAEGGGVR |
| rs2731672 | C | T | 1.79E-13 | 0.081 | 0.011 | 0.743 | 54.223 | 0.265 | ADSGEGDFXAEGGGVR |
| rs7061710 | C | G | 7.52E-14 | 0.045 | 0.006 | 0.228 | 55.149 | 0.248 | X-11786--methylcysteine |
| rs887829 | T | C | 3.00E-114 | 0.093 | 0.004 | 0.328 | 516.731 | 2.909 | X-11793--oxidized bilirubin |
| rs1115381 | C | T | 5.59E-15 | 0.033 | 0.004 | 0.738 | 59.972 | 0.297 | X-11793--oxidized bilirubin |
| rs28899194 | T | C | 5.04E-11 | 0.072 | 0.011 | 0.035 | 42.843 | 0.036 | X-11793--oxidized bilirubin |
| rs6760588 | C | A | 2.92E-12 | -0.030 | 0.004 | 0.252 | 48.675 | 0.235 | X-11793--oxidized bilirubin |
| rs7104849 | A | G | 3.26E-11 | 0.034 | 0.005 | 0.917 | 43.509 | 0.085 | 1-arachidonoylglycerophosphocholine |
| rs1692120 | A | G | 5.82E-12 | 0.019 | 0.003 | 0.459 | 48.483 | 0.308 | 1-arachidonoylglycerophosphocholine |
| rs174535 | C | T | 1.86E-94 | -0.056 | 0.003 | 0.340 | 431.716 | 2.476 | 1-arachidonoylglycerophosphocholine |
| rs12202350 | C | T | 5.07E-18 | -0.060 | 0.007 | 0.090 | 74.610 | 0.156 | Isobutyrylcarnitine |
| rs662138 | G | C | 4.98E-49 | -0.077 | 0.005 | 0.168 | 217.563 | 0.777 | Isobutyrylcarnitine |
| rs648253 | A | G | 6.02E-31 | -0.041 | 0.004 | 0.507 | 131.612 | 0.841 | Isobutyrylcarnitine |
| rs3798167 | T | G | 2.20E-22 | 0.043 | 0.004 | 0.206 | 96.843 | 0.405 | Isobutyrylcarnitine |
| rs9457843 | T | C | 1.32E-16 | 0.050 | 0.006 | 0.122 | 68.063 | 0.186 | Isobutyrylcarnitine |
| rs2404602 | G | A | 3.56E-13 | -0.026 | 0.004 | 0.560 | 51.760 | 0.326 | Isobutyrylcarnitine |
| rs603424 | A | G | 1.31E-14 | -0.037 | 0.005 | 0.185 | 58.459 | 0.225 | Palmitoleate (16:1n7) |
| rs4687717 | C | T | 1.07E-13 | -0.013 | 0.002 | 0.575 | 54.596 | 0.341 | Erythronate |
| rs6430553 | T | C | 6.29E-12 | -0.018 | 0.003 | 0.393 | 48.463 | 0.296 | X-12095--N1-methyl-3-pyridone-4-carboxamide |
| rs2160860 | T | A | 7.35E-11 | -0.012 | 0.002 | 0.386 | 44.444 | 0.269 | X-12100--hydroxytryptophan* |
| rs6804368 | G | A | 8.17E-21 | -0.022 | 0.002 | 0.429 | 84.793 | 0.531 | X-12244--N-acetylcarnosine |
| rs7775554 | C | A | 1.35E-15 | -0.019 | 0.002 | 0.472 | 62.674 | 0.399 | X-12244--N-acetylcarnosine |
| rs8002180 | C | T | 8.23E-23 | 0.024 | 0.002 | 0.280 | 99.168 | 0.511 | X-12244--N-acetylcarnosine |
| rs4148421 | C | T | 1.42E-10 | -0.015 | 0.002 | 0.554 | 40.641 | 0.257 | X-12244--N-acetylcarnosine |
| rs651007 | T | C | 6.34E-20 | 0.065 | 0.007 | 0.210 | 84.588 | 0.359 | ADpSGEGDFXAEGGGVR |
| rs601338 | A | G | 2.52E-11 | 0.041 | 0.006 | 0.432 | 44.373 | 0.278 | ADpSGEGDFXAEGGGVR |
| rs968567 | T | C | 2.84E-19 | 0.040 | 0.005 | 0.180 | 79.012 | 0.298 | 1-eicosatrienoylglycerophosphocholine |
| rs2271316 | G | C | 1.79E-23 | -0.113 | 0.011 | 0.618 | 100.532 | 0.607 | X-12441--12-hydroxyeicosatetraenoate (12-HETE) |
| rs6685187 | G | A | 2.88E-26 | 0.043 | 0.004 | 0.591 | 111.020 | 0.686 | X-12442--5,8-tetradecadienoate |
| rs721204 | A | G | 6.29E-16 | 0.047 | 0.006 | 0.112 | 66.551 | 0.169 | Octanoylcarnitine |
| rs5745515 | T | C | 8.24E-12 | 0.047 | 0.007 | 0.097 | 46.004 | 0.103 | Octanoylcarnitine |
| rs7552404 | G | A | 1.27E-77 | -0.075 | 0.004 | 0.302 | 347.823 | 1.873 | Octanoylcarnitine |
| rs5745542 | G | C | 2.17E-15 | 0.046 | 0.006 | 0.103 | 62.901 | 0.148 | Octanoylcarnitine |
| rs17304141 | G | T | 4.07E-22 | 0.142 | 0.015 | 0.021 | 92.919 | 0.050 | Octanoylcarnitine |
| rs6849801 | G | C | 1.60E-11 | 0.028 | 0.004 | 0.303 | 45.316 | 0.245 | Octanoylcarnitine |
| rs8396 | C | T | 1.38E-31 | -0.048 | 0.004 | 0.303 | 135.921 | 0.734 | Octanoylcarnitine |
| rs12141041 | C | T | 1.77E-12 | 0.025 | 0.004 | 0.531 | 49.390 | 0.314 | Alpha-hydroxyisovalerate |
| rs2403254 | T | C | 1.26E-30 | -0.041 | 0.004 | 0.551 | 129.075 | 0.816 | Alpha-hydroxyisovalerate |
| rs7552404 | G | A | 3.32E-43 | -0.056 | 0.004 | 0.302 | 188.560 | 1.015 | Decanoylcarnitine |
| rs17304141 | G | T | 1.87E-10 | 0.098 | 0.015 | 0.022 | 40.661 | 0.022 | Decanoylcarnitine |
| rs11722868 | G | A | 4.86E-14 | -0.031 | 0.004 | 0.693 | 57.908 | 0.315 | Decanoylcarnitine |
| rs8396 | C | T | 8.69E-38 | -0.053 | 0.004 | 0.303 | 169.000 | 0.913 | Decanoylcarnitine |
| rs174601 | T | C | 7.93E-16 | -0.034 | 0.004 | 0.340 | 64.000 | 0.367 | Stearidonate (18:4n3) |
| rs7778571 | G | A | 8.91E-12 | -0.045 | 0.007 | 0.246 | 46.281 | 0.219 | Epiandrosterone sulfate |
| rs474229 | T | G | 3.51E-17 | 0.049 | 0.006 | 0.602 | 71.957 | 0.441 | Epiandrosterone sulfate |
| rs11981478 | T | C | 2.80E-75 | -0.184 | 0.010 | 0.086 | 340.034 | 0.684 | Epiandrosterone sulfate |
| rs13222543 | T | C | 3.31E-47 | -0.347 | 0.024 | 0.020 | 207.790 | 0.106 | Epiandrosterone sulfate |
| rs6710726 | G | A | 1.27E-15 | -0.030 | 0.004 | 0.343 | 64.000 | 0.368 | X-12510--2-aminooctanoic acid |
| rs1318987 | A | C | 6.60E-24 | -0.055 | 0.005 | 0.157 | 102.609 | 0.347 | X-12510--2-aminooctanoic acid |
| rs17009399 | C | G | 1.56E-23 | -0.081 | 0.008 | 0.058 | 99.507 | 0.138 | X-12510--2-aminooctanoic acid |
| rs13538 | G | A | 7.89E-157 | -0.119 | 0.004 | 0.217 | 726.548 | 3.158 | X-12510--2-aminooctanoic acid |
| rs887829 | T | C | 1.79E-56 | 0.081 | 0.005 | 0.324 | 252.249 | 1.413 | Bilirubin (E,Z or Z,E) |
| rs174578 | A | T | 3.02E-24 | -0.027 | 0.003 | 0.341 | 104.669 | 0.601 | 1-arachidonoylglycerophosphoinositol |
| rs1871395 | G | A | 3.66E-31 | 0.040 | 0.003 | 0.159 | 134.970 | 0.461 | 1-arachidonoylglycerophosphoinositol |
| rs11045834 | T | C | 1.16E-10 | -0.017 | 0.003 | 0.291 | 43.256 | 0.228 | 1-arachidonoylglycerophosphoinositol |
| rs8736 | T | C | 8.45E-18 | -0.023 | 0.003 | 0.438 | 76.226 | 0.480 | 1-arachidonoylglycerophosphoinositol |
| rs8008020 | C | T | 5.89E-11 | 0.012 | 0.002 | 0.586 | 42.250 | 0.262 | Asparagine |
| rs2011069 | G | A | 1.56E-41 | -0.027 | 0.002 | 0.630 | 182.250 | 1.086 | Asparagine |
| rs2662314 | C | T | 4.86E-12 | -0.030 | 0.004 | 0.865 | 46.798 | 0.139 | Isovalerylcarnitine |
| rs11950562 | C | A | 2.31E-41 | -0.036 | 0.003 | 0.477 | 179.759 | 1.146 | Isovalerylcarnitine |
| rs17672041 | T | C | 1.31E-11 | 0.030 | 0.004 | 0.120 | 45.870 | 0.124 | Isovalerylcarnitine |
| rs9635324 | G | A | 1.89E-35 | -0.034 | 0.003 | 0.393 | 155.787 | 0.950 | Isovalerylcarnitine |
| rs272881 | A | G | 1.11E-10 | -0.021 | 0.003 | 0.617 | 42.053 | 0.254 | Stearoylcarnitine |
| rs588136 | T | C | 8.30E-12 | -0.029 | 0.004 | 0.796 | 46.430 | 0.193 | 1-stearoylglycerophosphoethanolamine |
| rs5030062 | C | A | 5.93E-13 | 0.072 | 0.010 | 0.385 | 51.552 | 0.312 | Bradykinin, des-arg(9) |
| rs4253311 | G | A | 3.56E-48 | 0.141 | 0.010 | 0.513 | 211.898 | 1.353 | Bradykinin, des-arg(9) |
| rs2731672 | C | T | 2.76E-23 | 0.169 | 0.017 | 0.743 | 98.827 | 0.482 | Bradykinin, des-arg(9) |
| rs2727271 | T | A | 2.74E-11 | -0.055 | 0.008 | 0.112 | 44.070 | 0.112 | X-12990--docosapentaenoic acid (n6-DPA) |
| rs5760492 | A | G | 2.34E-12 | -0.049 | 0.007 | 0.333 | 49.401 | 0.280 | Cysteine-glutathione disulfide |
| rs273914 | T | A | 3.04E-11 | -0.023 | 0.004 | 0.632 | 43.938 | 0.261 | Oleoylcarnitine |
| rs174578 | A | T | 6.12E-42 | -0.035 | 0.003 | 0.341 | 185.379 | 1.065 | 1-arachidonoylglycerophosphoethanolamine |
| rs4149056 | C | T | 2.66E-18 | 0.029 | 0.003 | 0.160 | 74.772 | 0.256 | 1-arachidonoylglycerophosphoethanolamine |
| rs10468017 | T | C | 1.29E-10 | 0.017 | 0.003 | 0.288 | 40.111 | 0.210 | 1-arachidonoylglycerophosphoethanolamine |
| rs12566232 | C | A | 5.69E-19 | 0.045 | 0.005 | 0.291 | 79.566 | 0.419 | X-13431--nonanoylcarnitine |
| rs1509821 | T | C | 5.28E-14 | 0.057 | 0.008 | 0.109 | 57.042 | 0.142 | X-13431--nonanoylcarnitine |
| rs4672574 | C | T | 1.99E-11 | -0.033 | 0.005 | 0.364 | 45.082 | 0.267 | X-13431--nonanoylcarnitine |
| rs3738934 | C | T | 1.21E-134 | 0.106 | 0.004 | 0.377 | 603.102 | 3.621 | X-13431--nonanoylcarnitine |
| rs2302539 | A | G | 1.37E-28 | 0.049 | 0.004 | 0.400 | 125.033 | 0.767 | X-13431--nonanoylcarnitine |
| rs2539861 | C | A | 2.21E-11 | -0.057 | 0.009 | 0.081 | 44.969 | 0.085 | X-13431--nonanoylcarnitine |
| rs2270968 | G | T | 1.31E-16 | -0.031 | 0.004 | 0.737 | 68.845 | 0.341 | Hydroxyisovaleroyl carnitine |
| rs13375749 | C | T | 2.80E-28 | -0.030 | 0.003 | 0.220 | 121.000 | 0.531 | Glutaroyl carnitine |
| rs715 | C | T | 3.50E-16 | -0.022 | 0.003 | 0.283 | 68.380 | 0.354 | Glutaroyl carnitine |
| rs17641971 | C | T | 3.35E-22 | 0.025 | 0.003 | 0.339 | 93.941 | 0.538 | Glutaroyl carnitine |
| rs2291449 | G | A | 1.44E-10 | 0.028 | 0.004 | 0.086 | 41.198 | 0.083 | Glutaroyl carnitine |
| rs8056893 | A | C | 2.23E-30 | -0.030 | 0.003 | 0.734 | 134.917 | 0.674 | Glutaroyl carnitine |
| rs246234 | G | C | 2.56E-14 | 0.020 | 0.003 | 0.704 | 58.581 | 0.312 | Glutaroyl carnitine |
| rs8012 | G | A | 6.36E-45 | -0.037 | 0.003 | 0.559 | 191.874 | 1.209 | Glutaroyl carnitine |
| rs896388 | A | G | 3.94E-11 | 0.031 | 0.005 | 0.077 | 43.504 | 0.079 | Glutaroyl carnitine |
| rs2070895 | A | G | 1.36E-18 | 0.030 | 0.003 | 0.208 | 76.305 | 0.321 | 1-palmitoylglycerophosphoethanolamine |
| rs10468017 | T | C | 3.33E-12 | 0.019 | 0.003 | 0.288 | 47.968 | 0.251 | 1-palmitoylglycerophosphoethanolamine |
| rs6663731 | T | A | 1.38E-14 | -0.062 | 0.008 | 0.114 | 59.347 | 0.154 | Tetradecanedioate |
| rs12317268 | G | A | 4.16E-60 | 0.101 | 0.006 | 0.166 | 264.325 | 0.935 | Tetradecanedioate |
| rs11045821 | A | G | 4.60E-30 | -0.074 | 0.007 | 0.164 | 128.561 | 0.451 | Tetradecanedioate |
| rs6663731 | T | A | 5.49E-11 | -0.045 | 0.007 | 0.118 | 42.912 | 0.114 | Hexadecanedioate |
| rs2857468 | T | A | 6.06E-11 | 0.062 | 0.009 | 0.918 | 42.805 | 0.082 | Hexadecanedioate |
| rs11045818 | A | G | 1.61E-32 | -0.073 | 0.006 | 0.161 | 143.214 | 0.495 | Hexadecanedioate |
| rs1871395 | G | A | 1.65E-52 | 0.089 | 0.006 | 0.167 | 229.086 | 0.816 | Hexadecanedioate |
| rs968567 | T | C | 2.14E-21 | 0.033 | 0.004 | 0.180 | 88.360 | 0.333 | Dihomo-linolenate (20:3n3 or n6) |
| rs6498540 | G | A | 2.55E-11 | -0.017 | 0.003 | 0.296 | 43.763 | 0.233 | Dihomo-linolenate (20:3n3 or n6) |
| rs12829704 | A | G | 9.49E-21 | -0.042 | 0.005 | 0.193 | 87.111 | 0.346 | Octadecanedioate |
| rs17806888 | C | T | 7.15E-11 | 0.022 | 0.003 | 0.119 | 43.405 | 0.117 | Succinylcarnitine |
| rs10988217 | G | A | 1.36E-18 | 0.016 | 0.002 | 0.612 | 74.504 | 0.452 | Succinylcarnitine |
| rs12899230 | C | T | 1.39E-11 | 0.025 | 0.004 | 0.079 | 47.075 | 0.087 | Succinylcarnitine |
| rs1472631 | G | A | 1.85E-88 | -0.037 | 0.002 | 0.508 | 413.444 | 2.641 | Succinylcarnitine |
| rs2729816 | C | T | 5.54E-33 | -0.031 | 0.003 | 0.781 | 144.925 | 0.634 | Succinylcarnitine |
| rs2729786 | A | C | 3.97E-11 | 0.024 | 0.004 | 0.099 | 43.707 | 0.100 | Succinylcarnitine |
| rs924135 | T | A | 1.52E-19 | 0.017 | 0.002 | 0.617 | 86.077 | 0.520 | Succinylcarnitine |
| rs2405522 | G | A | 4.49E-29 | 0.126 | 0.011 | 0.833 | 125.123 | 0.445 | Tryptophan betaine |
| rs10278040 | A | G | 1.17E-29 | -0.190 | 0.017 | 0.042 | 128.040 | 0.131 | 5alpha-androstan-3beta,17beta-diol disulfate |
| rs13222543 | T | C | 1.18E-20 | -0.263 | 0.028 | 0.020 | 86.979 | 0.044 | 5alpha-androstan-3beta,17beta-diol disulfate |
| rs2547231 | A | C | 3.35E-22 | 0.081 | 0.008 | 0.830 | 92.985 | 0.336 | 5alpha-androstan-3beta,17beta-diol disulfate |
| rs4149452 | T | C | 1.71E-12 | 0.052 | 0.007 | 0.263 | 49.760 | 0.246 | 5alpha-androstan-3beta,17beta-diol disulfate |
| rs4802397 | C | T | 1.09E-10 | 0.062 | 0.010 | 0.141 | 41.845 | 0.130 | 4-androsten-3beta,17beta-diol disulfate 1 |
| rs4149452 | T | C | 3.01E-23 | 0.077 | 0.008 | 0.266 | 98.213 | 0.490 | 4-androsten-3beta,17beta-diol disulfate 1 |
| rs296396 | C | T | 1.48E-92 | 0.175 | 0.009 | 0.835 | 415.970 | 1.466 | 4-androsten-3beta,17beta-diol disulfate 1 |
| rs7259671 | C | T | 3.83E-11 | -0.044 | 0.007 | 0.680 | 43.440 | 0.242 | 4-androsten-3beta,17beta-diol disulfate 1 |
| rs2762353 | G | A | 2.50E-13 | -0.030 | 0.004 | 0.546 | 53.183 | 0.337 | 4-androsten-3beta,17beta-diol disulfate 2 |
| rs4149056 | C | T | 1.33E-18 | 0.049 | 0.006 | 0.161 | 77.760 | 0.269 | 4-androsten-3beta,17beta-diol disulfate 2 |
| rs721204 | A | G | 2.22E-12 | 0.042 | 0.006 | 0.112 | 49.938 | 0.127 | Cis-4-decenoyl carnitine |
| rs17304141 | G | T | 5.93E-17 | 0.120 | 0.014 | 0.021 | 70.067 | 0.036 | Cis-4-decenoyl carnitine |
| rs11161521 | C | T | 1.22E-63 | -0.061 | 0.004 | 0.302 | 285.235 | 1.536 | Cis-4-decenoyl carnitine |
| rs8396 | C | T | 8.96E-13 | -0.026 | 0.004 | 0.304 | 50.142 | 0.271 | Cis-4-decenoyl carnitine |
| β, a ratio of changes in standard deviations; EA, effect allele; EAF, effect allele frequency; OA, other allele; SNP, single nucleotide polymorphism; SE, standard error. ^a^ F = β^2^/SE^2^, F > 10 means enough powerful of individual SNP [21] ^b^ R^2^ = 2×EAF×(1−EAF)×β^2^/SE^2^/N, N means sample size [22] | | | | | | | | | |

| **Supplementary Table 3** Documented SNPs with potential genetic correlation^*^ with known confounders of HNC | | | | | | |
| --- | --- | --- | --- | --- | --- | --- |
| **SNP** | **Pos (hg19)** | **EA** | **OA** | **Trait** | **β** | **P value** |
| rs601338 | chr19:49206674 | A | G | Alcohol intake frequency | -0.021 | 3.08E-09 |
| rs2141371 | chr2:27860258 | G | A | Alcohol intake frequency | -0.030 | 1.04E-13 |
| rs174538 | chr11:61560081 | A | G | Average weekly champagne plus white wine intake | 0.014 | 3.49E-08 |
| rs174550 | chr11:61571478 | C | T | Average weekly champagne plus white wine intake | 0.014 | 2.40E-08 |
| β, a ratio of changes in standard deviations; Chr, chromosome; EA, effect allele; OA, other allele; HNC, head and neck cancer; Pos, position based on GRCh37/hg19; SNP, single nucleotide polymorphism. ^*^ from the web tool PhenoScanner (version 2, http://www.phenoscanner.medschl.cam.ac.uk/) | | | | | | |

| **Supplementary Table 4** Nominal associations of genetically predicted another 23 blood metabolites with the risk of HNC based on MR method | | | | | | | | | | | | | | | | | | | | | | | | | |
| --- | --- | --- | --- | --- | --- | --- | --- | --- | --- | --- | --- | --- | --- | --- | --- | --- | --- | --- | --- | --- | --- | --- | --- | --- | --- |
| **Exposures** |  | **IVW** | | |  | **P_h_** |  | **P_p_** |  | **MR-Egger** | | |  | **Simple mode** | | |  | **Weighted median** | | |  | **Weighted mode** | | |  |
|  |  | **β** | **SE** | **P value** |  |  |  |  |  | **β** | **SE** | **P value** |  | **β** | **SE** | **P value** |  | **β** | **SE** | **P value** |  | **β** | **SE** | **P value** |  |
| 10-undecenoate (11:1n1) |  | 0.279 | 1.452 | 0.848 |  | 0.167 |  | NA |  | - | - | - |  | - | - | - |  | - | - | - |  | - | - | - |  |
| 3-dehydrocarnitine |  | 0.888 | 1.748 | 0.612 |  | 0.542 |  | 0.716 |  | 4.073 | 6.876 | 0.660 |  | 0.451 | 2.563 | 0.877 |  | 0.314 | 1.842 | 0.865 |  | 0.131 | 2.100 | 0.956 |  |
| 4-acetamidobutanoate |  | -2.273 | 1.820 | 0.212 |  | 0.301 |  | 0.439 |  | 3.436 | 4.992 | 0.616 |  | -0.860 | 2.430 | 0.757 |  | -1.659 | 1.810 | 0.360 |  | -1.453 | 2.011 | 0.545 |  |
| Arachidonate (20:4n6) |  | -0.798 | 1.403 | 0.569 |  | 0.188 |  | NA |  | - | - | - |  | - | - | - |  | - | - | - |  | - | - | - |  |
| Betaine |  | 3.425 | 2.105 | 0.104 |  | 0.423 |  | NA |  | - | - | - |  | - | - | - |  | - | - | - |  | - | - | - |  |
| Bilirubin (Z,Z) |  | -0.722 | 0.616 | 0.240 |  | 0.638 |  | 0.439 |  | -1.990 | 1.457 | 0.305 |  | 0.106 | 1.024 | 0.924 |  | -0.840 | 0.724 | 0.246 |  | -1.240 | 0.939 | 0.279 |  |
| Biliverdin |  | -0.748 | 1.383 | 0.589 |  | 0.171 |  | 0.923 |  | -1.584 | 7.134 | 0.861 |  | -2.207 | 1.968 | 0.379 |  | -1.689 | 1.341 | 0.208 |  | -2.316 | 2.156 | 0.395 |  |
| Carnitine |  | -1.567 | 3.471 | 0.652 |  | 0.558 |  | 0.335 |  | 13.234 | 12.254 | 0.393 |  | -5.358 | 6.092 | 0.444 |  | -3.151 | 4.124 | 0.445 |  | -5.358 | 5.902 | 0.431 |  |
| Glutaroyl carnitine |  | -0.822 | 0.871 | 0.345 |  | 0.992 |  | 0.995 |  | -0.851 | 4.456 | 0.856 |  | -1.233 | 1.417 | 0.418 |  | -1.098 | 1.028 | 0.286 |  | -1.240 | 1.320 | 0.384 |  |
| Glycine |  | 1.804 | 1.160 | 0.120 |  | 0.952 |  | 0.541 |  | -2.508 | 6.567 | 0.722 |  | 2.870 | 1.837 | 0.179 |  | 1.579 | 1.441 | 0.273 |  | 0.958 | 1.821 | 0.621 |  |
| Isobutyrylcarnitine ^a^ |  | -1.104 | 0.939 | 0.240 |  | 0.424 |  | 0.415 |  | -5.974 | 3.836 | 0.363 |  | -1.749 | 1.399 | 0.338 |  | -1.494 | 1.038 | 0.150 |  | -1.650 | 1.164 | 0.292 |  |
| Isovalerylcarnitine ^b^ |  | -0.504 | 1.206 | 0.676 |  | 0.739 |  | 0.902 |  | 1.498 | 12.954 | 0.927 |  | -0.040 | 1.723 | 0.983 |  | -0.355 | 1.329 | 0.789 |  | -0.288 | 1.551 | 0.870 |  |
| Kynurenine |  | -0.475 | 2.048 | 0.817 |  | 0.812 |  | NA |  | - | - | - |  | - | - | - |  | - | - | - |  | - | - | - |  |
| N-acetylornithine |  | -0.273 | 0.336 | 0.416 |  | 0.362 |  | 0.327 |  | -1.108 | 0.726 | 0.267 |  | -0.143 | 0.544 | 0.810 |  | -0.402 | 0.367 | 0.273 |  | -0.417 | 0.365 | 0.336 |  |
| Proline |  | 0.828 | 1.660 | 0.618 |  | 0.069 |  | 0.313 |  | -3.997 | 3.895 | 0.413 |  | 1.312 | 2.506 | 0.637 |  | 1.038 | 1.449 | 0.474 |  | 1.312 | 2.269 | 0.604 |  |
| Succinylcarnitine |  | 2.241 | 1.851 | 0.226 |  | 0.391 |  | 0.553 |  | 10.054 | 11.247 | 0.466 |  | 0.421 | 3.197 | 0.903 |  | 1.129 | 2.225 | 0.612 |  | 0.272 | 2.959 | 0.933 |  |
| Tryptophan |  | 1.921 | 6.836 | 0.779 |  | 0.610 |  | NA |  | - | - | - |  | - | - | - |  | - | - | - |  | - | - | - |  |
| Urate |  | -0.650 | 2.184 | 0.766 |  | 0.077 |  | 0.877 |  | 0.161 | 5.439 | 0.978 |  | -4.466 | 2.968 | 0.207 |  | -0.857 | 1.737 | 0.622 |  | -0.529 | 1.915 | 0.796 |  |
| X-03056--N-[3-(2-Oxopyrrolidin-1-yl)propyl]acetamide |  | 0.555 | 2.134 | 0.795 |  | 0.033 |  | 0.283 |  | 9.566 | 4.493 | 0.280 |  | -2.312 | 2.226 | 0.408 |  | -0.215 | 1.536 | 0.889 |  | -2.148 | 2.880 | 0.534 |  |
| X-11593--O-methylascorbate |  | 0.676 | 0.955 | 0.479 |  | 0.827 |  | 0.654 |  | 1.622 | 2.135 | 0.503 |  | 1.401 | 1.872 | 0.496 |  | 0.932 | 1.044 | 0.372 |  | 1.237 | 1.144 | 0.340 |  |
| X-12244--N-acetylcarnosine |  | 0.698 | 1.458 | 0.632 |  | 0.358 |  | 0.797 |  | -2.482 | 11.011 | 0.843 |  | -1.181 | 2.557 | 0.676 |  | 0.091 | 1.714 | 0.957 |  | -1.106 | 2.410 | 0.677 |  |
| X-12510--2-aminooctanoic acid |  | 0.378 | 1.132 | 0.739 |  | 0.787 |  | NA |  | - | - | - |  | - | - | - |  | - | - | - |  | - | - | - |  |
| X-13431--nonanoylcarnitine |  | -0.573 | 0.451 | 0.204 |  | 0.747 |  | 0.560 |  | -1.212 | 1.077 | 0.343 |  | -0.563 | 0.807 | 0.524 |  | -0.589 | 0.510 | 0.249 |  | -0.590 | 0.521 | 0.321 |  |
| β, a ratio of changes in standard deviations; HNC, head and neck cancer; IVM, inverse-variance-weighted method; MR, Mendelian randomisation; NA, not applicable; P_h_, P-value for heterogeneity based on Cochran’s Q statistic; P_p_, P-value for pleiotropy based on MR-Egger intercept; SE, standard error. ^a^ GWAS ID: met-a-573 ^b^ GWAS ID: met-a-652 | | | | | | | | | | | | | | | | | | | | | | | | | |

_
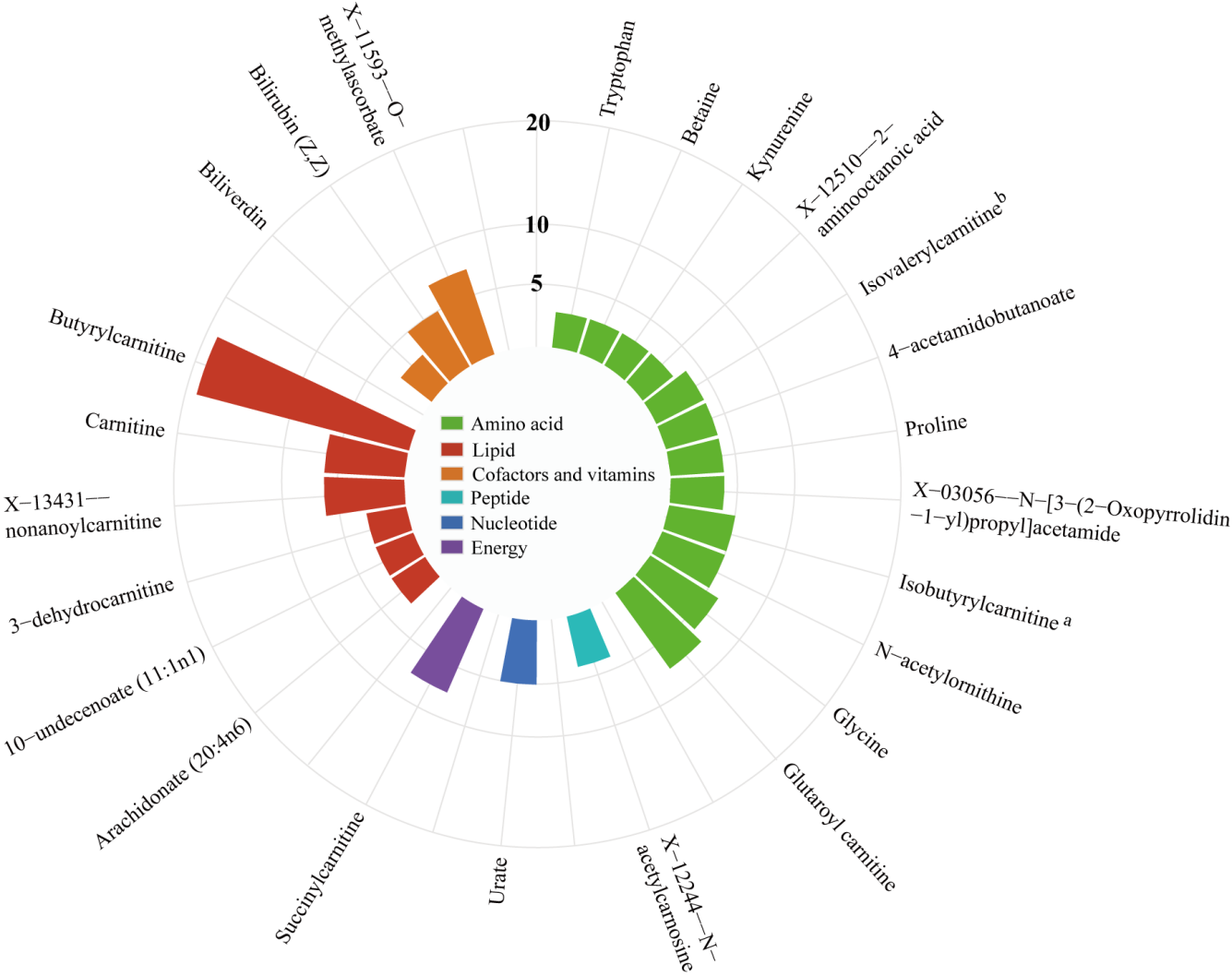
_

**Supplementary Figure 1** The final IVs of 122 SNPs associated with 24 human blood metabolites

_
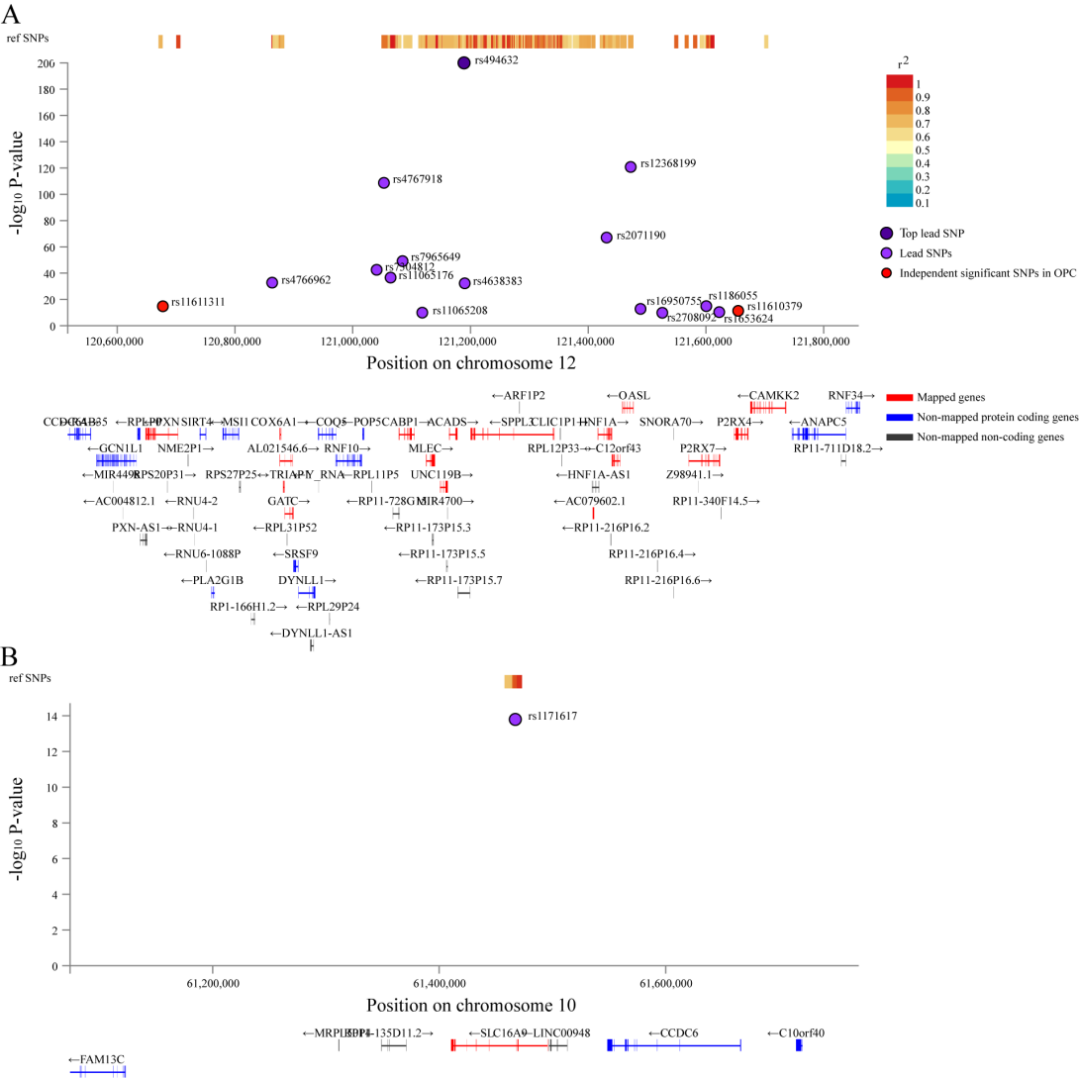
_

**Supplementary Figure 2** Regional plot for the risk loci strongly associated with butyrylcarnitine in (A) the chromosome 12 and (B) the chromosome 10 using FUMA
