# Supplementary material for: Causal association between blood metabolites and head and neck cancer: butyrylcarnitine identified as an associated trait for cancer risk and progression
Source: Hereditas. 2025 Mar 14;162:36. doi: 10.1186/s41065-025-00408-5 (PMC11907814; doi:10.1186/s41065-025-00408-5)
Supplement: Supplementary file 2 — Supplementary Material 2 [file 41065_2025_408_MOESM2_ESM.docx]

**STROBE-MR checklist of recommended items to address in reports of Mendelian randomization studies**^1^ ^2^

| **Item No.** | **Section** | **Checklist item** | **Page No.** | **Relevant text from manuscript** |
| --- | --- | --- | --- | --- |
| 1 | **TITLE and ABSTRACT** | Indicate Mendelian randomization (MR) as the study’s design in the title and/or the abstract if that is a main purpose of the study | 1-2 | Title: The blood metabolite butyrylcarnitine predicts the risk of tumorigenesis risk and promotes the development of head and neck cancer  Abstract: Methods |
|  | **INTRODUCTION** |  |  |  |
| 2 | **Background** | Explain the scientific background and rationale for the reported study. What is the exposure? Is a potential causal relationship between exposure and outcome plausible? Justify why MR is a helpful method to address the study question | 3-4 | In order to investigate the causality of human blood metabolites (exposure) on the risk of HNC (outcome), we conducted a two-sample MR analysis.  Recently, studies on blood metabolites have provided numerous biomarkers and established reliable prediction models for predicting the occurrence of tumors, such as cancers of the breast, prostate, kidney, and so on. However, little is known about the causality of blood metabolites and HNC.  Compared to conventional observational studies in the field of causal inference, MR is less susceptible to confounding factors and reverse causality bias |
| 3 | **Objectives** | State specific objectives clearly, including pre-specified causal hypotheses (if any). State that MR is a method that, under specific assumptions, intends to estimate causal effects | 5 | Hence, in this study, two-sample MR analysis was used to explore the causal relationship between blood metabolites and the occurrence of HNC, and the possible carcinogenic role of butyrylcarnitine in HNC cell line was verified by in vitro experiments, so as to provide references for guiding the risk prediction of HNC and the development of therapeutic targets. |
|  | **METHODS** |  |  |  |
| 4 | **Study design and data sources** | Present key elements of the study design early in the article. Consider including a table listing sources of data for all phases of the study. For each data source contributing to the analysis, describe the following: |  |  |
|  | a) | Setting: Describe the study design and the underlying population, if possible. Describe the setting, locations, and relevant dates, including periods of recruitment, exposure, follow-up, and data collection, when available. | 5-6 | All analyses were based on publicly available summary statistics. The study design was shown in Fig. 1 |
|  | b) | Participants: Give the eligibility criteria, and the sources and methods of selection of participants. Report the sample size, and whether any power or sample size calculations were carried out prior to the main analysis | 5-7 | All analyses were based on publicly available summary statistics. The the sources and methods of selection of participants were described in “Summary statistics of explore and outcome” |
|  | c) | Describe measurement, quality control and selection of genetic variants | 7 | Selection of genetic variant |
|  | d) | For each exposure, outcome, and other relevant variables, describe methods of assessment and diagnostic criteria for diseases | 7-8 | Summary statistics of explore and outcome |
|  | e) | Provide details of ethics committee approval and participant informed consent, if relevant | 5 | This study was based on publicly available data from GWASs. All studies had been approved by appropriate ethical review committee in their respective studies. |
| 5 | **Assumptions** | Explicitly state the three core IV assumptions for the main analysis (relevance, independence and exclusion restriction) as well assumptions for any additional or sensitivity analysis | 5-6 | The fundamental tenets of MR analysis, namely relevance, independence, and exclusion restriction, were upheld throughout this investigation, which mean (1) selected genetic variants are robustly associated with the exposure; (2) the genetic variants remain independent of potential confounding factors; (3) their sole influence on the outcome is mediated through the exposure. |
| 6 | **Statistical methods: main analysis** | Describe statistical methods and statistics used | 6-11 | Summary statistics of explore and outcome and Statistical Analysis |
|  | a) | Describe how quantitative variables were handled in the analyses (i.e., scale, units, model) | NA | NA |
|  | b) | Describe how genetic variants were handled in the analyses and, if applicable, how their weights were selected | 7 | Selection of genetic variant |
|  | c) | Describe the MR estimator (e.g. two-stage least squares, Wald ratio) and related statistics. Detail the included covariates and, in case of two-sample MR, whether the same covariate set was used for adjustment in the two samples | 8-9 | Mendelian randomization analysis |
|  | d) | Explain how missing data were addressed | NA | NA |
|  | e) | If applicable, indicate how multiple testing was addressed | NA | NA |
| 7 | **Assessment of assumptions** | Describe any methods or prior knowledge used to assess the assumptions or justify their validity | 5 | The fundamental tenets of MR analysis, namely relevance, independence, and exclusion restriction, were upheld throughout this investigation, which mean (1) selected genetic variants are robustly associated with the exposure; (2) the genetic variants remain independent of potential confounding factors; (3) their sole influence on the outcome is mediated through the exposure. |
| 8 | **Sensitivity analyses and additional analyses** | Describe any sensitivity analyses or additional analyses performed (e.g. comparison of effect estimates from different approaches, independent replication, bias analytic techniques, validation of instruments, simulations) | 8-9 | Mendelian randomization analysis |
| 9 | **Software and pre-registration** |  |  |  |
|  | a) | Name statistical software and package(s), including version and settings used | 12-13 | R software assisted in performing all statistical analysis (v4.0.4, https://www.r-project.org/). MR analysis was conducted by using the “TwoSampleMR” package. |
|  | b) | State whether the study protocol and details were pre-registered (as well as when and where) | NA | NA |
|  | **RESULTS** |  |  |  |
| 10 | **Descriptive data** |  |  |  |
|  | a) | Report the numbers of individuals at each stage of included studies and reasons for exclusion. Consider use of a flow diagram | 5 | A brief flow of the study is displayed in Fig. 1. |
|  | b) | Report summary statistics for phenotypic exposure(s), outcome(s), and other relevant variables (e.g. means, SDs, proportions) | 7 | The related information on datasets used is presented in Supplemental [Table 1](https://www.ncbi.nlm.nih.gov/pmc/articles/PMC9294168/#SM1). |
|  | c) | If the data sources include meta-analyses of previous studies, provide the assessments of heterogeneity across these studies | NA | The heterogeneity assessments across meta-analyses has been described by Shin et al. The summary statistics utilized in this study were provided by Integrative Epidemiology Unit (IEU) open GWAS project (https://gwas.mrcieu.ac.uk/). |
|  | d) | For two-sample MR:  i.  Provide justification of the similarity of the genetic variant-exposure associations between the exposure and outcome samples  ii.  Provide information on the number of individuals who overlap between the exposure and outcome studies | 6 | They were all European populations.  There was little overlap in selection of participants between the exposure group and the outcome group. |
| 11 | **Main results** |  |  |  |
|  | a) | Report the associations between genetic variant and exposure, and between genetic variant and outcome, preferably on an interpretable scale | 14-15 | Genetically determined blood metabolites and risk of HNC |
|  | b) | Report MR estimates of the relationship between exposure and outcome, and the measures of uncertainty from the MR analysis, on an interpretable scale, such as odds ratio or relative risk per SD difference | 14-15 | Genetically determined blood metabolites and risk of HNC |
|  | c) | If relevant, consider translating estimates of relative risk into absolute risk for a meaningful time period | 14-15 | Genetically determined blood metabolites and risk of HNC |
|  | d) | Consider plots to visualize results (e.g. forest plot, scatterplot of associations between genetic variants and outcome versus between genetic variants and exposure) | 14-15 | Fig. 2 |
| 12 | **Assessment of assumptions** |  |  |  |
|  | a) | Report the assessment of the validity of the assumptions | 14-15 | Genetically determined blood metabolites and risk of HNC |
|  | b) | Report any additional statistics (e.g., assessments of heterogeneity across genetic variants, such as *I^2^*, Q statistic or E-value) | 15 | According to Cochran’s Q test, our findings revealed no significant heterogeneity (Q = 14.161, p = 0.587), strengthening the reliability and consistency of our study outcomes. |
| 13 | **Sensitivity analyses and additional analyses** |  |  |  |
|  | a) | Report any sensitivity analyses to assess the robustness of the main results to violations of the assumptions | 15 | To ascertain the robustness of the IVW analysis outcomes, a sensitivity analysis was conducted. According to Cochran’s Q test, our findings revealed no significant heterogeneity (Q = 14.161, p = 0.587), strengthening the reliability and consistency of our study outcomes. |
|  | b) | Report results from other sensitivity analyses or additional analyses | 15 | MR-Egger regression method also indicated no existence of directional pleiotropy (intercept = -0.007, p = 0.886). |
|  | c) | Report any assessment of direction of causal relationship (e.g., bidirectional MR) | 15 | Moreover, the MR Steiger test demonstrated an absence of evidence of reverse causality (psteiger < 0.01). |
|  | d) | When relevant, report and compare with estimates from non-MR analyses | 16-17 | Functional annotations of risk SNPs |
|  | e) | Consider additional plots to visualize results (e.g., leave-one-out analyses) | 15 | It showed that no single SNP strongly altered the overall effect of butyrylcarnitine on risk of HNC, indicating the reliability and stability of our analysis according to “leave-one-out” method (Fig. 2). |
|  | **DISCUSSION** |  |  |  |
| 14 | **Key results** | Summarize key results with reference to study objectives | 18 | Among the 258 known blood metabolites, our findings pinpoint butyrylcarnitine as a potential causal mediator with plausible causal implications in the development of HNC. Namely, genetically predicted high butyrylcarnitine level is associated with increased risks of HNC. |
| 15 | **Limitations** | Discuss limitations of the study, taking into account the validity of the IV assumptions, other sources of potential bias, and imprecision. Discuss both direction and magnitude of any potential bias and any efforts to address them | 23-24 | Several limitations warrant consideration when interpreting our findings. Firstly, the genetic variants analyzed in this study were derived from a single cohort, and their effects on HNC risk have not been validated in independent cohorts. Moreover, while cis-SNPs for a biomarker present a robust MR evidence, caution must be exercised when utilizing MR scanning biomarkers due to the unavailability of cis instruments to corroborate MR signals stemming from trans-genetic variations [9]. Lastly, we present evidence underscoring the significance of butyrylcarnitine in HNC etiology and the roles of butyrylcarnitine on HNC cells in vitro; however, this does not exclude the possibility of other metabolites within the butyrylcarnitine pathway exerting biological effects on HNC. Further investigations are warranted to comprehensively explore each constituent of the pathway and elucidate the intricate mechanisms underlying the correlation between cis-SNPs and HNC, both in vitro and in vivo. |
| 16 | **Interpretation** |  |  |  |
|  | a) | Meaning: Give a cautious overall interpretation of results in the context of their limitations and in comparison with other studies | 18-22 | Despite the scarcity of epidemiological data examining the significance of butyrylcarnitine in HNC, previous studies have identified circulating butyrylcarnitine as one of the metabolites exhibiting mutual associations with breast cancer [43]. Nonetheless, a comprehensive investigation encompassing multiple cancer types including breast, colorectal, endometrial, gallbladder, kidney, prostate cancer, and hepatocellular carcinoma revealed no significant association between butyrylcarnitine and cancer risk in cancer-specific univariate analyses, while noteworthy findings did emerge from the univariate pooled analysis and the data-shared lasso analysis, warranting further exploration [8]. In addition, Hatae et al. indicated that butyrylcarnitine are linked to antitumor immune responses to the PD-1 blockade therapy which may be due to FAO-mediated T cell dysfunction |
|  | b) | Mechanism: Discuss underlying biological mechanisms that could drive a potential causal relationship between the investigated exposure and the outcome, and whether the gene-environment equivalence assumption is reasonable. Use causal language carefully, clarifying that IV estimates may provide causal effects only under certain assumptions | 16-17,22-23 | OASL/rs16950755 as a potential causal gene for HNC |
|  | c) | Clinical relevance: Discuss whether the results have clinical or public policy relevance, and to what extent they inform effect sizes of possible interventions | 16-17 | To analyze the mRNA expression levels of local genes influenced by cis-SNPs, we conducted gene expression discrepancies between patients diagnosed with HNC and controls using data from the GEO database. We speculated that the involvement of rs16950755 in the occurrence of HNC might be accomplished by regulating the expression levels of OALS gene. |
| 17 | **Generalizability** | Discuss the generalizability of the study results (a) to other populations, (b) across other exposure periods/timings, and (c) across other levels of exposure | 18-22 | Shin et al. conducted a large GWAS on human blood metabolites to date in 2014, providing significant insights into the genetic underpinnings of blood metabolomics [11]. Recently, Smith-Byrne et al. employed a bidirectional MR analysis to assess the causal relationship between blood metabolites and lung cancer, and found an inverse association of elevated blood isovalerylcarnitine with lung cancer risk [12]. Zhong et al. demonstrated that metabolite X-21849 was linked to pancreatic ductal adenocarcinoma risk, while Wang et al. revealed the mediating role of high-density lipoprotein cholesterol and acetate in the development of breast cancer |
|  | **OTHER INFORMATION** |  |  |  |
| 18 | **Funding** | Describe sources of funding and the role of funders in the present study and, if applicable, sources of funding for the databases and original study or studies on which the present study is based | 26 | Funding |
| 19 | **Data and data sharing** | Provide the data used to perform all analyses or report where and how the data can be accessed, and reference these sources in the article. Provide the statistical code needed to reproduce the results in the article, or report whether the code is publicly accessible and if so, where | 26-27 | Availability of data and materials |
| 20 | **Conflicts of Interest** | All authors should declare all potential conflicts of interest | 27 | Competing interests |

This checklist is copyrighted by the Equator Network under the Creative Commons Attribution 3.0 Unported (CC BY 3.0) license.

1. Skrivankova VW, Richmond RC, Woolf BAR, Yarmolinsky J, Davies NM, Swanson SA, et al. Strengthening the Reporting of Observational Studies in Epidemiology using Mendelian Randomization (STROBE-MR) Statement. JAMA. 2021;under review.

2. Skrivankova VW, Richmond RC, Woolf BAR, Davies NM, Swanson SA, VanderWeele TJ, et al. Strengthening the Reporting of Observational Studies in Epidemiology using Mendelian Randomisation (STROBE-MR): Explanation and Elaboration. BMJ. 2021;375:n2233.
